# Silica Induction of Diverse Inflammatory Proteome in Lungs of Lupus-Prone Mice Quelled by Dietary Docosahexaenoic Acid Supplementation

**DOI:** 10.3389/fimmu.2021.781446

**Published:** 2022-01-21

**Authors:** Lichchavi D. Rajasinghe, Melissa A. Bates, Abby D. Benninghoff, Kathryn A. Wierenga, Jack R. Harkema, James J. Pestka

**Affiliations:** ^1^ Department of Food Science and Human Nutrition, Michigan State University, East Lansing, MI, United States; ^2^ Institute for Integrative Toxicology, Michigan State University, East Lansing, MI, United States; ^3^ Department of Animal, Dairy and Veterinary Sciences, School of Veterinary Medicine, Utah State University, Logan, UT, United States; ^4^ Department of Biochemistry and Molecular Biology, Michigan State University, East Lansing, MI, United States; ^5^ Department of Pathobiology and Diagnostic Investigation, Michigan State University, East Lansing, MI, United States; ^6^ Department of Microbiology and Molecular Genetics, Michigan State University, East Lansing, MI, United States

**Keywords:** systemic lupus erythematosus, chemokine, metalloproteinase, TNF superfamily, omega-3 fatty acid, autoimmunity, inflammation

## Abstract

Repeated short-term intranasal instillation of lupus-prone mice with crystalline silica (cSiO_2_) induces inflammatory gene expression and ectopic lymphoid neogenesis in the lung, leading to early onset of systemic autoimmunity and rapid progression to glomerulonephritis. These responses are suppressed by dietary supplementation with the ω-3 polyunsaturated fatty acid docosahexaenoic acid (DHA). Here, we tested the hypothesis that dietary DHA supplementation suppresses cSiO_2_-induced inflammatory proteins in bronchoalveolar alveolar lavage fluid (BALF) and plasma of lupus-prone mice. Archived tissue fluid samples were used from a prior investigation in which 6 wk-old lupus-prone female NZBWF1 mice were fed isocaloric diets containing 0 or 10 g/kg DHA for 2 wks and then intranasally instilled with 1 mg cSiO_2_ or vehicle once weekly for 4 wks. Cohorts were terminated at 1, 5, 9 or 13 wk post-instillation (PI). BALF and plasma from each cohort were analyzed by high density multiplex array profiling of 200 inflammatory proteins. cSiO_2_ time-dependently induced increases in the BALF protein signatures that were highly reflective of unresolved lung inflammation, although responses in the plasma were much less robust. Induced proteins in BALF included chemokines (*e.g.*, MIP-2, MCP-5), enzymes (*e.g.*, MMP-10, granzyme B), adhesion molecules (*e.g.*, sE-selectin, sVCAM-1), co-stimulatory molecules (*e.g.*, sCD40L, sCD48), TNF superfamily proteins (*e.g.*, sTNFRI, sBAFF-R), growth factors (*e.g.*, IGF-1, IGFBP-3), and signal transduction proteins (*e.g.*, MFG-E8, FcgRIIB), many of which were blocked or delayed by DHA supplementation. The BALF inflammatory proteome correlated positively with prior measurements of gene expression, pulmonary ectopic lymphoid tissue neogenesis, and induction of autoantibodies in the lungs of the control and treatment groups. Ingenuity Pathway Analysis (IPA) revealed that IL-1β, TNF-α, and IL-6 were among the top upstream regulators of the cSiO_2_-induced protein response. Furthermore, DHA’s effects were associated with downregulation of cSiO_2_-induced pathways involving i) inhibition of ARE‐mediated mRNA decay, ii) bacterial and viral pattern recognition receptor activation, or iii) TREM1, STAT3, NF-κB, and VEGF signaling and with upregulation of PPAR, LXR/RXR and PPARα/RXRα signaling. Altogether, these preclinical findings further support the contention that dietary DHA supplementation could be applicable as an intervention against inflammation-driven autoimmune triggering by cSiO_2_ or potentially other environmental agents.

## Introduction

Systemic lupus erythematosus (lupus), a chronic autoimmune disease predominantly affecting young women of child-bearing age, is caused by the loss of immunological tolerance resulting from yet poorly understood interactions between an individual’s genome and the environment (1, 2). Initial onset of this disease typically involves unresolved inflammation and incomplete clearance of dead cells, accumulation of self-antigens, and autoantibody production, leading to the formation of immune complexes. Tissue deposition of these complexes fosters cytokine and chemokine production, infiltration of mononuclear effector cells, and ultimately, cell death. Collectively, these pathologic effects promote systemic inflammation and tissue injury, adversely affecting multiple organs including kidney, skin, heart, lung, and brain (3). Frequent recurrent cycles of flaring and remission in lupus leads to permanent organ damage that, without treatment, can manifest as severe glomerulonephritis and end-stage renal disease (kidney failure). Lupus management includes therapies based on immunosuppression, lymphocyte depletion, and cytokine/chemokine neutralization with monoclonal antibodies or receptor antagonists (4). Limitations of existing treatments include heterogeneity of individual patient symptoms, diversity of responses to therapeutics, adverse side effects leading to permanent organ damage, and high expense leading to financial burden on the patient. Thus, there is clear need for alternative safe and low-cost interventions for lupus.

Environment can influence onset and progression of autoimmunity in individuals with a genetic predisposition towards lupus (5). Notably, crystalline silica dust (cSiO_2_) exposure in construction, mining, and ceramics industries has been epidemiologically associated with lupus and other autoimmune diseases (6–9). Induction of autoimmunity can be recapitulated at the preclinical level by introduction of cSiO_2_ dust particles into the lungs of mice predisposed to lupus (10–14). Our laboratory has found in lupus-prone female NZBWF1 hybrid mice that, following four repeated weekly intranasal instillations with cSiO_2_, latency of glomerulonephritis onset is reduced by three months (15, 16). At the mechanistic level, airway instillation of cSiO_2_ causes the persistent build-up of particle-bearing monocytes/macrophages, release of proinflammatory cytokines and chemokines, recruitment of neutrophils and macrophages into the alveolar space, programmed and necrotic cell death, and accumulation of nuclear and cytoplasmic debris (17). These events facilitate perivascular accumulation of T-cells, B-cells, and IgG autoantibody-producing plasma cells, leading to formation of ectopic lymphoid tissues (ELT) in the lung. The centrality of the lung in cSiO_2_-triggered lupus onset and progression in NZBWF1 mice provides a unique window into potential mechanisms for and interventions against autoimmune pathogenesis in humans exposed to this and other respirable particles in the environment.

Diet is another environmental factor that can influence onset and progression of autoimmunity, with lipids being of particular importance. Consumption of marine ω-3 polyunsaturated fatty acids (PUFAs) can ameliorate chronic inflammatory and autoimmune diseases (18) and extend human lifetime (19). Mechanisms underlying ω-3 attenuating effects include i) influencing membrane function by altering lipid rafts and signaling, ii) moderating gene expression by repressing/activating transcription factors, iii) competition with ω-6 PUFA for metabolic enzyme binding sites and iv) serving as substrates for formation of pro-resolving metabolites [reviewed in (18, 20, 21)]. Unfortunately, Western diets contain large amounts plant- and animal-derived fats, therefore skewing tissue phospholipid fatty acid content overwhelmingly towards the more proinflammatory ω-6 PUFAs and away from the anti-inflammatory ω-3 PUFAs (22, 23). However, this imbalance can be overcome by eating fish or dietary fish/microalgal oil supplements containing the ω-3 PUFAs docosahexaenoic acid (C22:6 ω-3; DHA) and eicosapentaenoic acid (C20:5 ω-3; EPA). Relevant to the present study, dietary supplementation with ω-3 PUFA-rich fish oil inhibits inflammatory gene expression, production of autoantibodies, glomerulonephritis, and/or death in numerous murine preclinical lupus models (24–29), with DHA-enriched fish oil being most efficacious (30, 31). Consistent with these mouse studies, several clinical investigations support the assertion that increased ω-3 PUFA consumption might benefit patients with lupus (21, 32–34).

To ascertain how ω-3 PUFAs influence environment-triggered lupus flaring, Bates and coworkers (16) compared cSiO_2_-triggered autoimmune disease onset and progression in autoimmune-prone female NZBWF1 mice fed control diets or diets containing DHA at the realistic human caloric equivalent of 2 or 5 g/d. After 2 wk on these dietary regimens, mice were treated weekly for 4 wk with intranasally instilled cSiO_2_ (1 mg) and then sacrificed 1, 5, 9, and 13 wk. DHA supplementation dose-dependently reduced cSiO_2_-triggered B- and T-cell, follicular dendritic cell, and IgG^+^ plasma cell accumulation in the lungs along with development of glomerulonephritis. Subsequently, tissues from the aforementioned study were analyzed for 800 immune-related genes using targeted multiplex platform (35). Intriguingly, pulmonary transcriptome signatures of cSiO_2_-treated mice fed control diet were indicative of progressive increases of genes associated with inflammation, innate/adaptive immunity, chemokines, type 1 interferon (IFN) responses, and antigen processing. Remarkably, DHA supplementation dose-dependently attenuated these responses, suggesting that consumption of this fatty acid impedes gene responses that play a central role in inflammation, ectopic lymphoid tissue neogenesis, and autoimmunity.

It is not yet known how modulation of cSiO_2_-induced gene expression by DHA influences the inflammatory proteome. We therefore conducted a targeted proteomic analysis of archived tissue fluids from the Bates et al. study (16) to test the hypothesis that short-term repeated pulmonary exposures of lupus-prone mice to cSiO_2_ induces inflammatory proteins in alveolar fluid and plasma, which are suppressed by DHA consumption. The results establish that cSiO_2_ elicited a diverse inflammatory proteome in the lung that was consistent with unresolved inflammation and pulmonary ectopic lymphoid neogenesis and, furthermore, dietary DHA supplementation markedly quelled this robust inflammatory protein response.

## Materials And Methods

### Experimental Design

**Figure 1:**
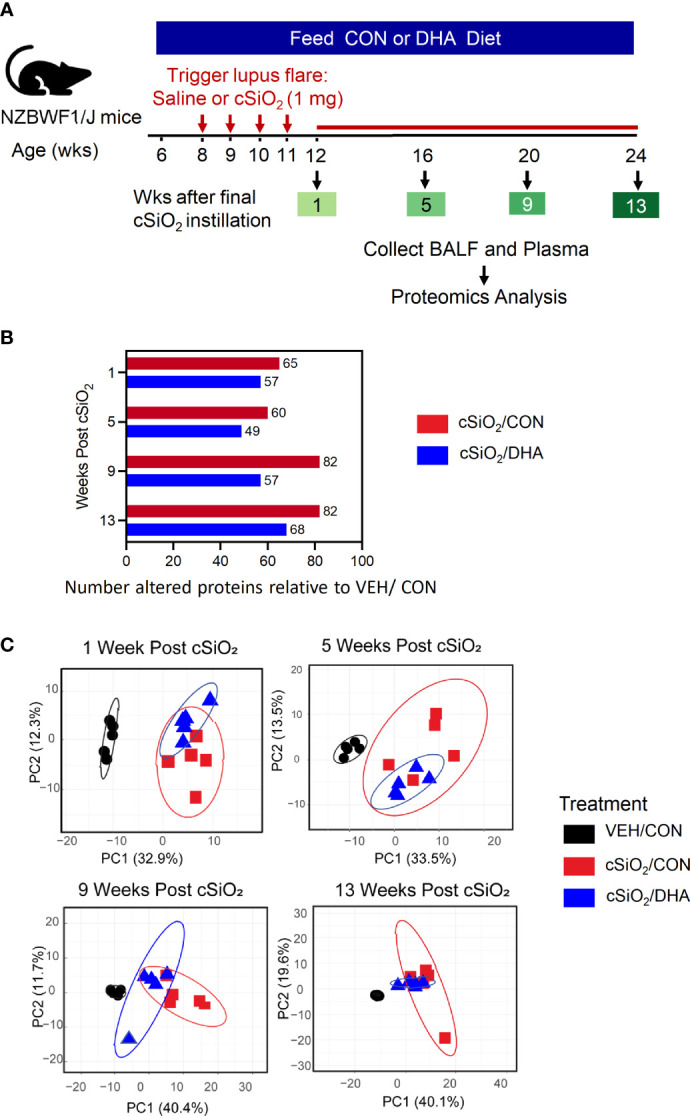
Experimental design and inflammatory protein analysis of BALF. **(A)** Feeding of control (CON) or DHA diets was initiated in 6 wk-old female NZBWF1 mice. Two wk later, groups were intranasally instilled with 1 mg cSiO_2_ or vehicle weekly for 4 wk. Animals were sacrificed at 12, 16, 20, or 24 wk of age, corresponding to 1, 5, 9, or 13 wk post final instillation (PI), respectively. Plasma and BALF were collected and analyzed for inflammatory proteins by high throughput multiplexed array. **(B)** cSiO_2_ induced fewer proteins in BALF of mice fed DHA diet over time than those fed control diet. **(C)** PCA of differentially expressed protein in BALF of VEH/CON, cSiO_2_/CON and cSiO_2_/DHA over time. Ellipses indicate 95% confidence intervals.

This study employed archived tissues from our prior investigation (16) that was approved by the Institutional Animal Care and Use Committee at Michigan State University (AUF #01/15-021-00). Briefly, as depicted in **Figure 1A**, 6-wk old female lupus-prone NZBWF1 mice (Jackson Laboratories, Bar Harbor, ME) were fed control (CON) diet containing 10 g/kg corn oil and 60 g/kg high-oleic safflower oil (Hain Pure Food, Boulder, CO), whereas the intervention diet (DHA) included 10 g/kg corn oil, 35 g/kg high-oleic safflower oil and 25 g/kg microalgal oil containing 40% DHA (DHASCO, DSM Nutritional Products, Columbia MD). Resulting diets had 0 or 10 g/kg DHA, respectively, which were calorically equivalent to human doses of 0 and 5 g per day, respectively. After 2 wk maintenance on designated diets, mice were anesthetized with 4% isoflurane and instilled intranasally with 1.0 mg cSiO_2_ (1.5-2.0 μm average particle size, U.S. Silica, Berkeley Springs, WV) in 25 μl PBS or 25μl PBS vehicle (VEH) alone. Afterwards, mice were fed assigned diets for the experiment duration. Cohorts (n=8/group) were terminated at 1, 5, 9, and 13 wk post-instillation (PI) of the final cSiO_2_ dose and bronchoalveolar lavage fluid (BALF), blood, and organs were collected, processed, and stored at -80^0^C as described previously (16). These specified times correspond with cSiO_2_-induced histopathologic, transcriptomic, and autoimmune effects in NZBWF1 mice (16, 35, 36).

### Targeted Inflammatory Proteomic Analysis by High Density Microarray ELISA

Selected samples (n=5/group) of BALF from 1, 5, 9, and 13 wk PI and plasma from 5, 9, and 13 wk PI were analyzed by RayBiotech (Norcross, GA) for the expression of 200 immune-related proteins using the Quantibody Array Q4000 (catalog #QAM-CAA-4000). Inflammation-associated proteins on the array were identified and functionally classified by RayBiotech as: i) chemokines, ii) adhesion molecules, iii) co-stimulatory molecules, iv) enzymes, v) signal transduction proteins, vi) TNF superfamily, vii) growth factors, and viii) cytokines (**Supplementary Table 1**). Briefly, BALF and plasma were diluted 3-fold and incubated for 2 h on glass arrays containing immobilized analyte-specific capture antibodies. Following incubation and washing, a cocktail of analyte-specific biotinylated detection antibodies was added and incubated for 2 h. After washing, arrays were incubated with Cy3-labeled streptavidin. A GenePix 4000B Microarray Scanner (Molecular Devices, Sunnyvale, CA) was used for fluorescent image acquisition. Resultant images were then digitized using GenePix software (Molecular Devices). Fluorescent intensities were acquired in quadruplicate for each sample and readings averaged to obtain mean fluorescent intensity (MFI). Signal to noise ratios (SNRs) were determined by comparing MFI to background signal for a specified analyte. SNRs greater than 2.0 were deemed positive for analyte signal above background.

### Data Analysis

Heat mapping with unsupervised hierarchical clustering (HCC) and Principal Component Analysis (PCA) were visualized using ClustVis (37). For heat mapping, normalized and unit variance-scaled Z-scores were depicted.


Z=(x−mean)/stdev.

Values were centered by rows with imputation used for missing value estimation and rows were clustered using Euclidean distance and Ward linkage. Initial statistical analyses included pairwise Student’s *t*-tests for all proteins to determine difference in expression for cSiO_2_-exposed mice provided CON or DHA diet to the vehicle control (VEH/CON). Following this exploratory analysis, select proteins were further examined by scatter plot (Prism v. 8.3.0, GraphPad, San Diego, CA). The robust regression and outlier removal (ROUT) method was employed to discern outliers, which were excluded from further analysis (Q = 0.5%). Data were analyzed by two-way ANOVA for experimental factors time point and treatment and their interaction, with *post-hoc* Tukey HSD multiple comparisons test to determine the effect of treatment at each time point. If individual groups failed to pass at least one normality for Gaussian distributions tests (Shapiro-Wilk, Kolmogorov-Smirnov, Anderson-Darling, D’Agostino-Pearson omnibus), data were log-transformed.

Spearman rank correlation analyses were performed using *cor* and *corrplot* functions in R (www.R-project.org) or using Prism (GraphPad). A significant correlation was inferred when *ρ* > 0.5 or <-0.5 and *p*<0.05. To corroborate protein expression data, Spearman rank correlations were performed between protein expression data from this study and gene expression data previously reported for corresponding animals (35), with both data sets represented as log_2_ fold-change in expression levels relative to the VEH/CON group. Log_2_ fold-changes in protein expression (expressed as MFI) relative to the VEH/CON group were calculated and correlated to the corresponding gene expression values. Spearman rank correlations for gene-protein interactions were determined using Microsoft Excel and bar graphs generated using GraphPad Prism.

Spearman rank correlations were also used to relate protein expression Z-scores with histological endpoints that included CD45R^+^ (B cells), CD3^+^ (T cells), CD21/35^+^ (follicular dendritic cells), and lymphoid aggregates, expressed as percent of lung area analyzed, which are collectively indicative of ectopic lymphoid neogenesis (16). For correlations to autoantibody production, signal intensities for individual autoantibodies (Ab-score) were used (36).

### IPA Analysis

Protein interactions and functional networks in the data set were determined using Ingenuity Pathway Analysis (IPA, QIAGEN Inc., Redwood City, CA). Calculated log_2_ fold-change protein values (sample MFI relative to mean MFI for VEH/CON group) for each protein were submitted for IPA core analysis with the protein’s UniProt identifiers. Protein expression was compared against its corresponding gene/protein expression data in the Ingenuity Pathway Knowledge Base. Canonical pathways and upstream regulator analysis tools in IPA core analysis were used at default setting to explore cSiO_2_ triggered immune-related pathways and predict expression of upstream regulators. The results from canonical pathways and upstream regulators analysis for different treatments groups were compared using comparison tool in IPA and visualized with GraphPad Prism. Proteins in data set with Z-scores ≥ 2 or ≤−2 with an overlapping p-value <0.05 were considered to be significantly activated or significantly inhibited, respectively, for all analyses or predictions.

## Results

### Induction of Diverse Inflammatory Proteome by cSiO_2_ in BALF Was Influenced by DHA Supplementation

Repeated intranasal instillations of cSiO_2_ in CON-fed lupus-prone NZBWF1 induced increased expression of a diverse group of proteins in BALF relative to baseline over time that were highly reflective of unresolved inflammation in the lung, with significant elevations in 65, 60, 82, and 82 proteins being observed at 1, 5, 9, and 13 wk PI, respectively (**Figure 1B**). DHA feeding reduced the number of proteins elevated at each timepoint, with cSiO_2_-treated DHA-fed mice exhibiting elevations in 57, 49, 57, and 68 proteins at 1, 5, 9, and 13 wk PI, respectively. PCA analysis further indicated that inflammatory proteomes in the BALF of cSiO_2_/CON and cSiO_2_/DHA mice at 1, 5, 9, and 13 wk PI were overlapping but different from those of VEH/CON mice (**Figure 1C**).

**Figure 2:**
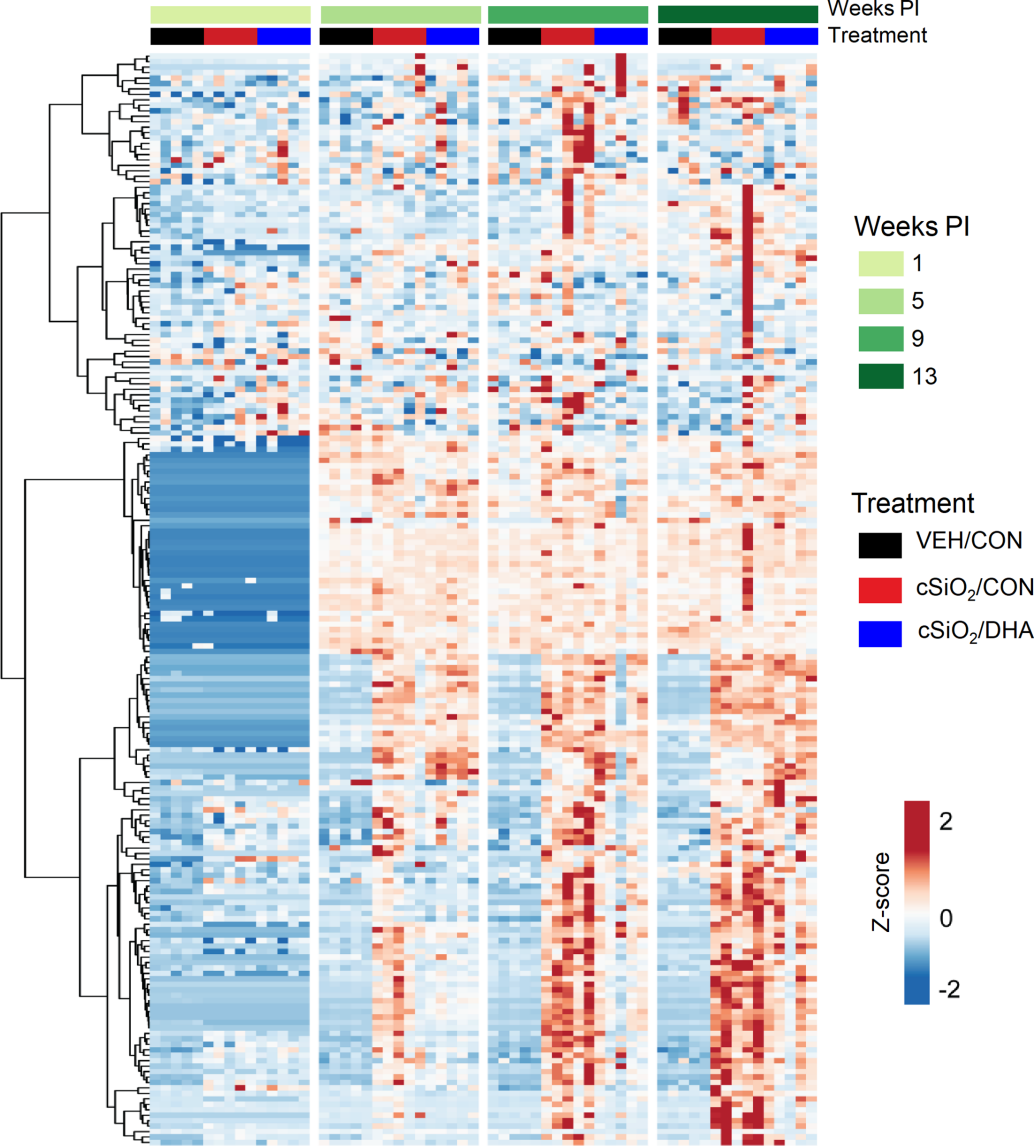
DHA intake modulates cSiO_2_-induced inflammatory protein responses in BALF. Heat map with unsupervised clustering (Euclidian distance method) depicting unit variance-scaled mean fluorescent intensities (MFIs) for 203 proteins. Black, red, and blue bars above the heatmap indicate the VEH/CON, cSiO_2_/CON, and cSiO_2_/DHA groups, respectively, at 1, 5, 9, or 13 wk PI.

Heat mapping revealed that cSiO_2_ treatment induced numerous chemokines (*e.g.*, MIP-2, MCP-5), enzymes (*e.g.*, MMP-10, granzyme B), adhesion molecules (*e.g.*, E-selectin, VCAM-1), co-stimulatory molecules (e.g., CD40L, CD48), TNF superfamily proteins (*e.g.*, TNFRI, BAFF-R), growth factors (*e.g.*, IGF-1, IGFBP-3), and signal transduction proteins (*e.g.*, MFG-E8, FcgRIIB) in the BALF (**Figure 2** and **Supplementary File 1**). Most of these were upregulated beginning at 5 wk PI and continued to increase over time, while time-matched VEH control groups remained unchanged or slightly increased. A small subset of cSiO_2_-induced inflammatory proteins in CON-fed mice were modestly elevated at 9 wk PI and remained so at 13 wk PI. DHA supplementation suppressed or delayed release of many cSiO_2_-induced proteins in BALF, particularly at 5 and 9 wk PI but less so at wk 13 suggesting some diminishment of DHA’s ameliorative effects. Spontaneous production of some proteins was also observed at 5, 9 or 13 wk PI in VEH control groups compared to earlier time points. The influence of time and treatment on specific protein families is described in further detail and illustrated in heat maps below.

### Chemokines Were Robustly Induced by cSiO_2_ in BALF and Suppressed by DHA Feeding While Cytokines Were Minimally Affected

**Figure 3:**
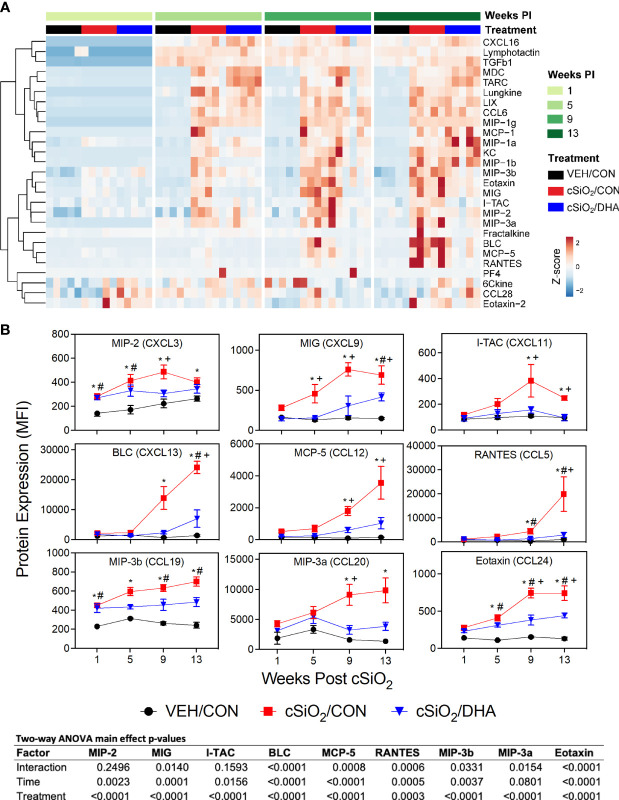
Consumption of DHA suppresses cSiO_2_-triggered chemokine expression in BALF. **(A)** Heat maps with unsupervised clustering (Euclidian distance method) depict unit variance-scaled mean fluorescent intensities (MFIs) for chemokine expression measured in the BALF. Black, red, and blue bars above the heatmap indicate the VEH/CON, cSiO_2_/CON, and cSiO_2_/DHA groups, respectively, at 1, 5, 9, or 13 wk PI. Scale bar values reflect the range of variance-stabilized MFIs, which were centered across rows. **(B)** DHA feeding suppressed selected cSiO_2_-induced chemokines in BALF over time. MFIs were obtained using the microarray panel for VEH- or cSiO_2_-exposed mice fed CON or DHA diet. Data for select proteins were analyzed by two-way ANOVA for main effects of timepoint and treatment with *post-hoc* Tukey HSD multiple comparison tests to determine effects of treatment at each time point. Data are mean ± SEM. Main effect p-values for timepoint, treatment, and the interaction of these factors are shown below the plots. Symbols indicate significant differences (p ≤ 0.05) as follows: * for cSiO_2_/CON vs. VEH/CON; # for cSiO_2_/DHA vs. VEH/CON; and + for cSiO_2_/CON vs. cSiO_2_/DHA.

cSiO_2_ elicited vigorous and diverse chemokine responses in BALF of CON-fed mice (**Figure 3A**). Effects were detected as early as 1 wk PI (MIP-2 and MIP-3b), whereas induction of others were not manifested until 5 wk PI (MIG, eotaxin-1) or 9 wk PI (MCP-5, RANTES, MIP-3a, I-TAC, BLC) (**Figure 3B**). DHA supplementation suppressed induction of these chemokines, particularly at wk 9 and 13 PI (**Figures 3A, B**). In contrast to robust chemokine responses, cSiO_2_ instillation did not have a large effect on BALF cytokines, with only modest upregulation being observed for IL-1α at 1 wk PI, IL-2Ra and IL-6 at 5 wk PI, and IL-17E and IL-17F at wk 9 PI (**Supplementary Figure 1**). Of these, only IL-2RA, IL-17E, and IL-17F induction were suppressed by DHA feeding.

### cSiO_2_-Triggered Elevations of Enzymes, Adhesion Molecules, and Co-Stimulatory Molecules in BALF Were Inhibited by DHA Feeding

**Figure 4:**
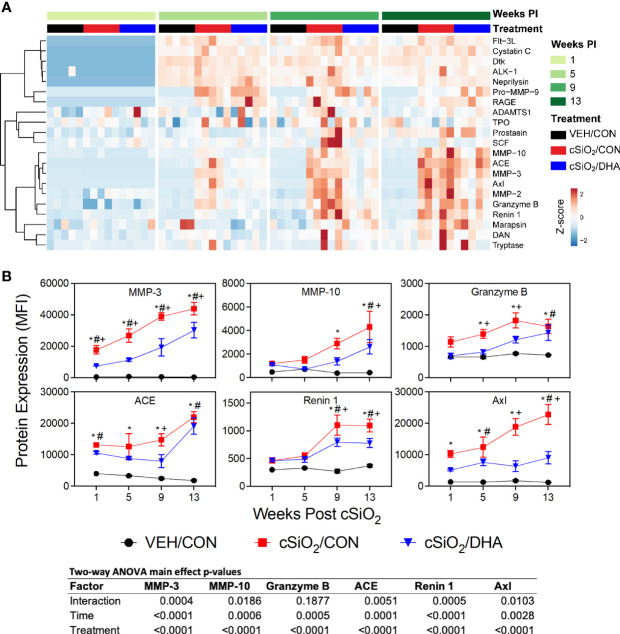
DHA supplementation inhibits cSiO_2_-stimulated release of inflammation-associated enzymes into the BALF. **(A)** Heat map with unsupervised clustering (Euclidian distance method) depicts unit variance-scaled mean fluorescent intensities (MFIs) for enzyme expression measured in the BALF. Black, red and blue bars above the heat map indicate the VEH/CON, cSiO_2_/CON, and cSiO_2_/DHA groups, respectively, at 1, 5, 9, or 13 wk PI. Scale bar values reflect the range of variance-stabilized MFIs, which were centered across rows. **(B)** DHA diet suppresses cSiO_2_ induction of selected enzymes in BALF over time. MFIs were obtained using the microarray panel for VEH- or cSiO_2_-exposed mice fed CON or DHA diets. Data for select proteins were analyzed by two-way ANOVA for main effects of timepoint and treatment with *post-hoc* Tukey HSD multiple comparison tests to determine effects of treatment at each time point. Data are mean ± SEM. Main effect p-values for timepoint, treatment, and the interaction of these factors are shown below the plots. Symbols indicate significant differences (p ≤ 0.05) as follows: * for cSiO_2_/CON vs. VEH/CON; # for cSiO_2_/DHA vs. VEH/CON; and + for cSiO_2_/CON vs. cSiO_2_/DHA.

**Figure 5:**
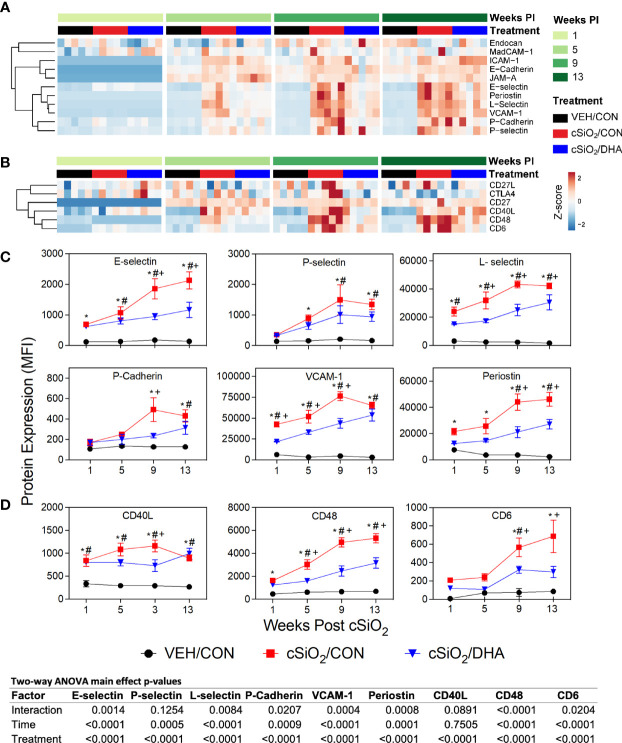
DHA consumption suppresses cSiO_2_-induced adhesion and co-stimulatory molecule release into the BALF. Heat maps with unsupervised clustering (Euclidian distance method) depict unit variance-scaled mean fluorescent intensities (MFIs) for **(A)** adhesion and **(B)** co-stimulatory molecules expression measured in the BALF. Black, red and blue bars above the heat map indicates the VEH/CON, cSiO_2_/CON, and cSiO_2_/DHA groups, respectively, at 1, 5, 9, or 13 wk PI. Scale bar values reflect the range of variance-stabilized MFIs, which were centered across rows. **(C, D)** Effects of DHA induction of selected **(C)** adhesion and **(D)** co-stimulatory molecules by cSiO_2_ in BALF over time. MFIs were obtained using the microarray panel for VEH- or cSiO_2_-exposed mice fed CON or DHA diets. Data for select proteins were analyzed by two-way ANOVA for main effects of timepoint and treatment with *post-hoc* Tukey HSD multiple comparison tests to determine effects of treatment at each time point. Data are mean ± SEM. Main effect p-values for timepoint, treatment, and the interaction of these factors are shown below the plots. Symbols indicate significant differences (p ≤ 0.05) as follows: * for cSiO_2_/CON vs. VEH/CON; # for cSiO_2_/DHA vs. VEH/CON; and + for cSiO_2_/CON vs. cSiO_2_/DHA.

cSiO_2_ triggered expression of diverse enzymes in BALF including MMP-3, ACE, and Axl at wk 1 PI, granzyme B at 5 wk PI, and MMP-10 and renin at 9 wk PI (**Figures 4A, B**). These responses were inhibited or delayed in cSiO_2_-treated DHA-fed mice. cSiO_2_ exposure further induced early and marked release of soluble adhesion molecules into the BALF of CON-fed mice as demonstrated by elevated sE-selectin, sL-selectin, sP-cadherin, sVCAM-1, and periostin at 1 wk PI and P-selectin at 5 wk PI in the BALF (**Figures 5A, C**). Increased soluble costimulatory molecules were also evident in CON/cSiO_2_ mice by 1 wk PI (CD40L, CD48) or 9 wk PI (CD6) (**Figures 5B, D**). While relative MFIs of these soluble adhesion and costimulatory molecules rose over time, these progressive increases were significantly delayed by DHA supplementation (**Figures 5B–D**).

### DHA Supplementation Slowed TNF Superfamily Elevation but Differentially Influenced Signal Transduction Molecule Release in BALF in Response to cSiO_2_

Instilling the lungs of CON-fed mice with cSiO_2_ also induced release of several soluble TNF superfamily members into BALF that were evident as early at 1 wk PI (TNFRI, TNFRII, and CD40) or later at 9 wk PI (BAFF-R, OX40L, and TRAIL) (**Supplementary Figure 2**). TNF superfamily responses were markedly delayed by feeding DHA. cSiO_2_ also elicited increases in soluble signal transduction molecules in BALF that included MFG-E8, FcgRIIB, and galectin-1 at 1 wk PI, leptin and MBL-2 at 5 wk PI, and resistin and B7-1 at 9 wk PI (**Supplementary Figure 3**). While DHA supplementation delayed leptin, FcgRIIB, galectin-1, and B7-1 responses, it enhanced MFG-E8 release.

### Rapid Induction of Diverse Growth Factor Proteins by cSiO2 Was Suppressed by DHA Consumption

**Figure 6:**
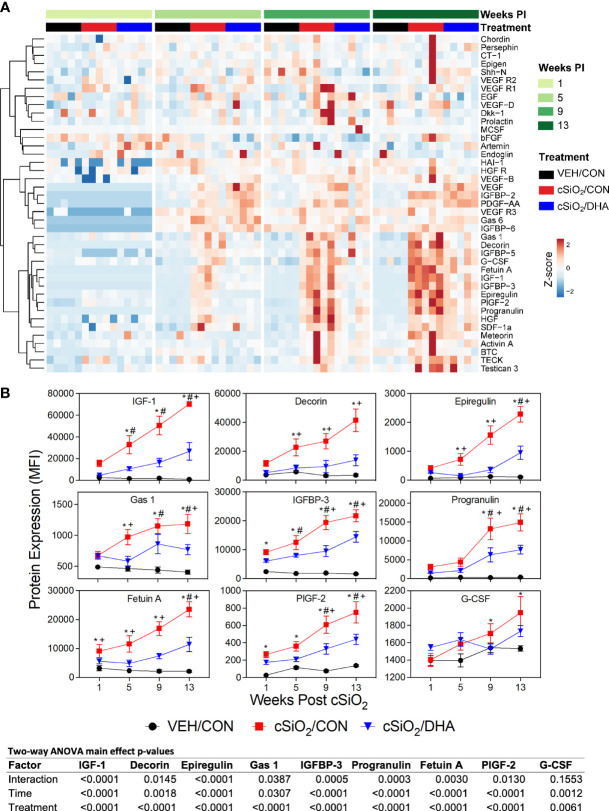
cSiO_2_-induced growth factor expression in BALF suppressed by intake of DHA diet. **(A)** Heat maps with unsupervised clustering (Euclidian distance method) depict unit variance-scaled mean fluorescent intensities (MFIs) for growth factor expression measured in the BALF. Black, red and blue bars above heat maps indicate the VEH/CON, cSiO_2_/CON, and cSiO_2_/DHA groups, respectively, at 1, 5, 9, or 13 wk PI. Scale bar values reflect the range of variance-stabilized MFIs, which were centered across rows. **(B)** Effect of DHA diet on cSiO_2_ induction of selected growth factors in BALF over time. MFIs were obtained using the microarray panel for VEH- or cSiO_2_-exposed mice fed CON or DHA diets. Data for select proteins were analyzed by two-way ANOVA for main effects of timepoint and treatment with *post-hoc* Tukey HSD multiple comparison tests to determine effects of treatment at each time point. Data are mean ± SEM. Main effect p-values for timepoint, treatment, and the interaction of these factors are shown below the plots. Symbols indicate significant differences (p ≤ 0.05) as follows: * for cSiO_2_/CON vs. VEH/CON; # for cSiO_2_/DHA vs. VEH/CON; and + for cSiO_2_/CON vs. cSiO_2_/DHA.

cSiO_2_ instillation triggered elevations in IGF-1, IGFBP-3, decorin, epiregulin, Gas-1, fetuin A, PIGF-2 by 1 or 5 wk PI, and, additionally, progranulin and G-CSF by wk 9 PI in CON-fed mice, whereas all but one of the growth factor responses were markedly suppressed in DHA-fed mice (**Figures 6A, B**).

### BALF Protein Responses Correlate Positively With Gene Expression and Autoimmune Pathogenesis in the Lung

**Figure 7:**
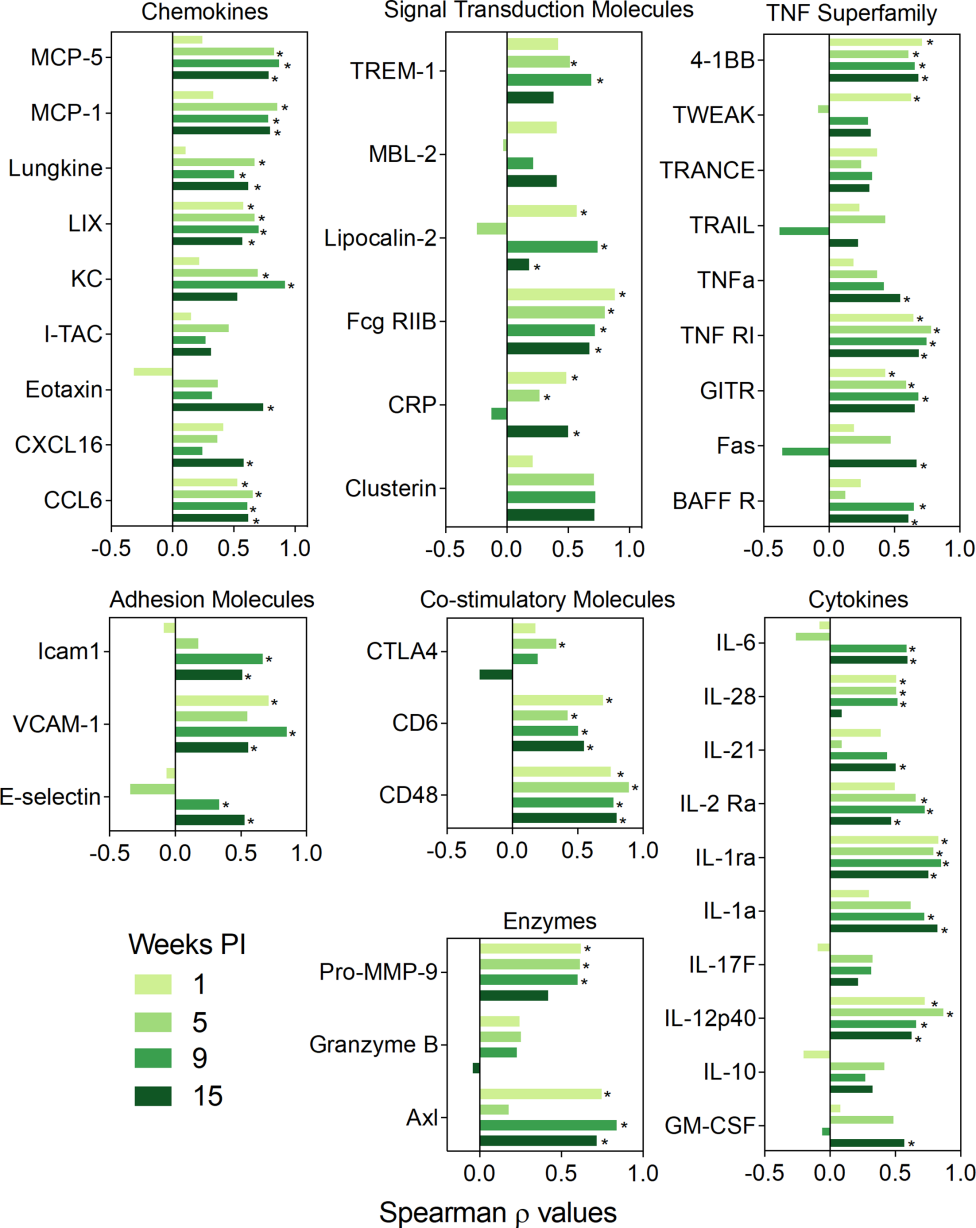
BALF protein responses positively correlate with corresponding lung gene expression. Spearman ρ values for individual classes of proteins, namely co-stimulatory molecules, adhesion molecules, chemokines, enzymes, signal transduction proteins, growth factors, TNF superfamily proteins, and cytokines were calculated by correlating with corresponding gene expression in individual mice from all experimental groups. Plots are separated by timepoint. Gene expression data were obtained previously using the NanoString Immune Profiling gene panel (35). Bar graph depicting the values of the Spearman correlation coefficient. Light green, moderately light green, green and dark green bars indicate 1, 5, 9, or 13 wk PI, respectively. Significant correlation values (p ≤ 0.05) are designated with *.

Where data sets overlapped, expression of proteins in the BALF correlated positively with the genes that encode them, as determined by mRNA expression of lung-associated genes assessed in our previous study (35) (**Figure 7**). These associations were strongest at 9 wk PI but also observed at 5 and 13 wk PI, albeit to a lesser extent. Specific proteins included chemokines (CCL6, CXCL16, eotaxin-1, I-TAC, KC, LIX, lungkine, MCP-1, MCP-5), cytokines (GM-CSF, IL-10, IL-12p40, IL-17F, IL-1a, IL-1ra, IL-2 Ra, IL-21, IL-28, IL-6), enzymes (Axl, granzyme B, Pro-MMP-9), adhesion molecules (E-selectin, VCAM-1, Icam1), co-stimulatory molecules (CD48, CD6, CTLA4), TNF superfamily proteins (BAFFR, Fas, GITR, TNFRI, TNFα, TRAIL, TRANCE, TWEAK, 4-1BB), and signal transduction proteins (clusterin, CRP, Fcg RIIB, lipocalin-2, MBL-2, TREM1). Thus, mRNA expression in the lung was reasonably predictive of inflammatory proteins in the alveolar fluid.

**Figure 8:**
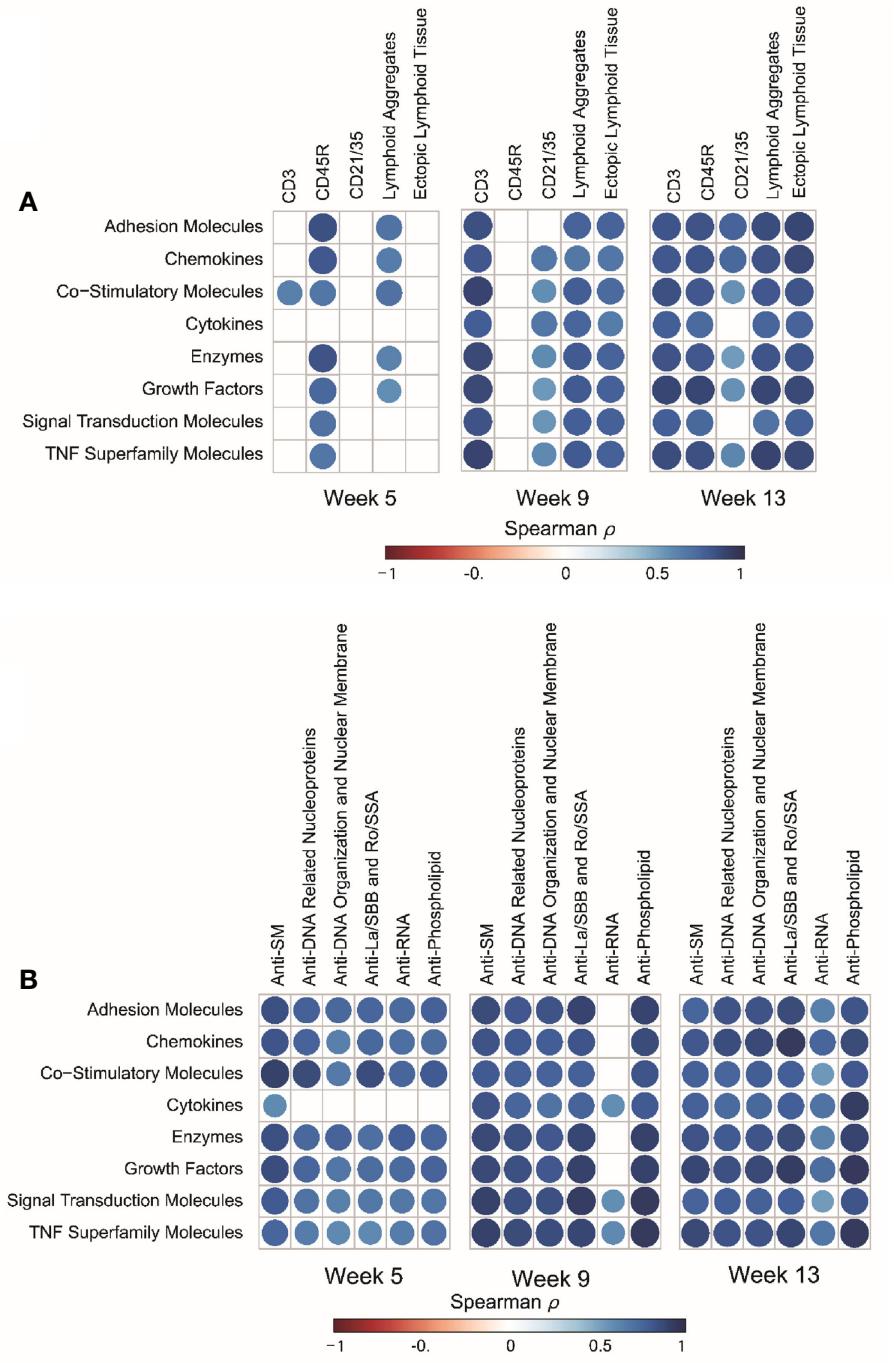
cSiO_2_-induced inflammatory proteins in BALF positively correlate with ectopic lymphoid tissue (ELT) development and IgG autoantibody responses. **(A)** For all cSiO_2_-treated groups, spearman ρ values were calculated by correlating Z scores for protein groups with markers for ELT development assessed in lung tissues of mice collected 5, 9, or 13 wk PI. Markers for ELT development in lung tissues of mice measured as non-continuous histopathology scores of lymphoid aggregates, ectopic lymphoid tissue and the percentage of positively stained tissues (CD3^+^, CD45R^+^, and CD21/35^+^) as described previously (16). **(B)** For all cSiO_2_-treated groups, Spearman ρ values were calculated by correlating Z scores with Σ IgG Ab-score values assessed in BALF of mice collected 5, 9, or 13 wk PI as previously reported (36). Σ Ab-score data calculated as the sum of autoantibodies belonging to a particular category. Significant correlation values (p ≤ .05) are represented as shaded circles; non-significant correlations are indicated by blank cells.

BALF protein responses for individual mice strongly correlated with histopathologic scoring of ELT development and with quantitative morphometry of CD45R^+^ B-cells, CD3^+^ T-cells, and CD21/35^+^ follicular dendritic cells in lungs of cSiO_2_-exposed mice, with strongest associations being observed at 13 wk PI (**Figure 8A**). Moreover, protein responses in individual mice correlated positively with IgG autoantibodies associated with Sm antigens, DNA related nucleoproteins, DNA organization and nuclear membrane proteins, La/SBB antigens, Ro/SSA antigens, RNA antigens, and phospholipids at 5, 9, and 13 wk PI (**Figure 8B**).

### DHA Supplementation Interferes With Regulatory Pathways for cSiO_2_-Triggered Inflammatory Protein Expression

**Figure 9:**
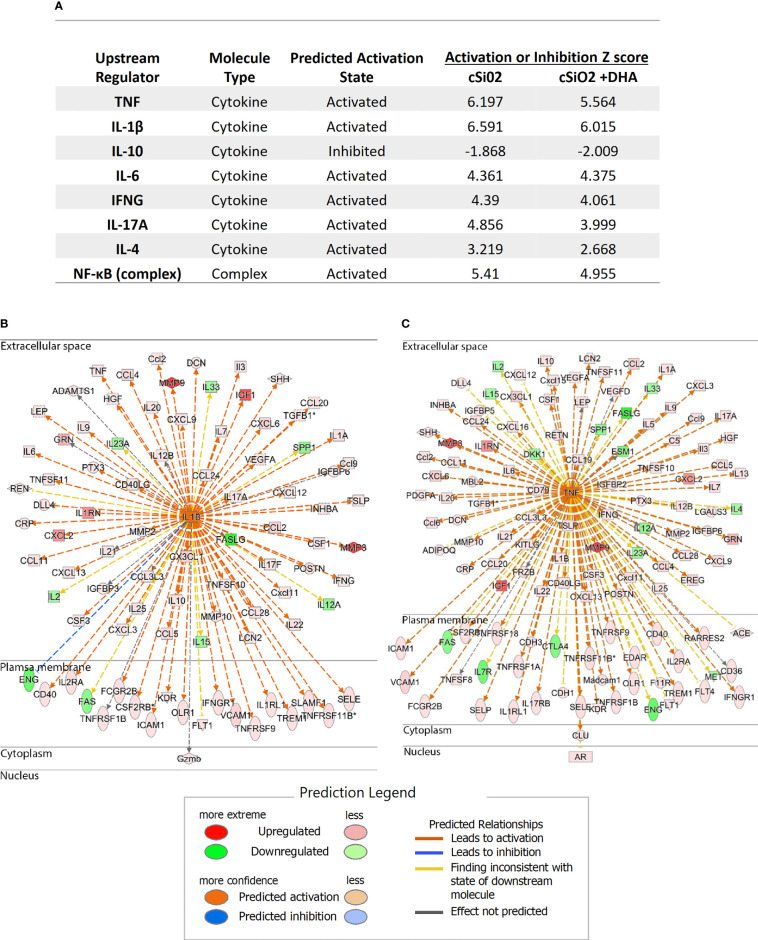
DHA supplementation negatively influences major upstream regulators of cSiO_2_-induced inflammatory protein expression. **(A)** Top upstream regulators present in cSiO_2_/CON and cSiO_2_/DHA treatments in BALF were predicted by IPA. Positive Z-score indicates activation, and negative Z-score indicates inhibition. Targets of **(B)** IL-1β and **(C)** TNF-α present in data set. Up-regulated and down-regulated proteins are highlighted in red and green, respectively, and the color depth is correlated to the fold change. Orange and blue dashed lines with arrows indicate indirect activation and inhibition, respectively. Yellow and gray dashed lines with arrows depict inconsistent effects and no prediction, respectively.

Protein expression data were subjected to IPA to identify key upstream molecules regulating the inflammatory response of the lung following cSiO_2_ and DHA treatment. Based on activation Z-scores, IL-1β, TNF-α, INFγ, IL-6, IL-17A, IL-4, and NF-κB complexes were the top upstream inflammatory proteome regulators predicted to be activated following cSiO_2_ instillation, whereas IL-10 was predicted to be inhibited following treatment with cSiO_2_ (**Figure 9A**). Importantly, feeding DHA reduced activation Z-scores associated with cSiO_2_-induced TNF-α, IL-1β, INFγ, IL-17A, IL-4, IL-10 and NF-κB complex, while increasing the inhibition Z-score for IL-10 (**Figure 9A**). **Figures 9B, C** reflect diverse cSiO_2_-induced inflammatory proteome predicted to be downstream of IL-1β and TNF-α, respectively.

**Figure 10:**
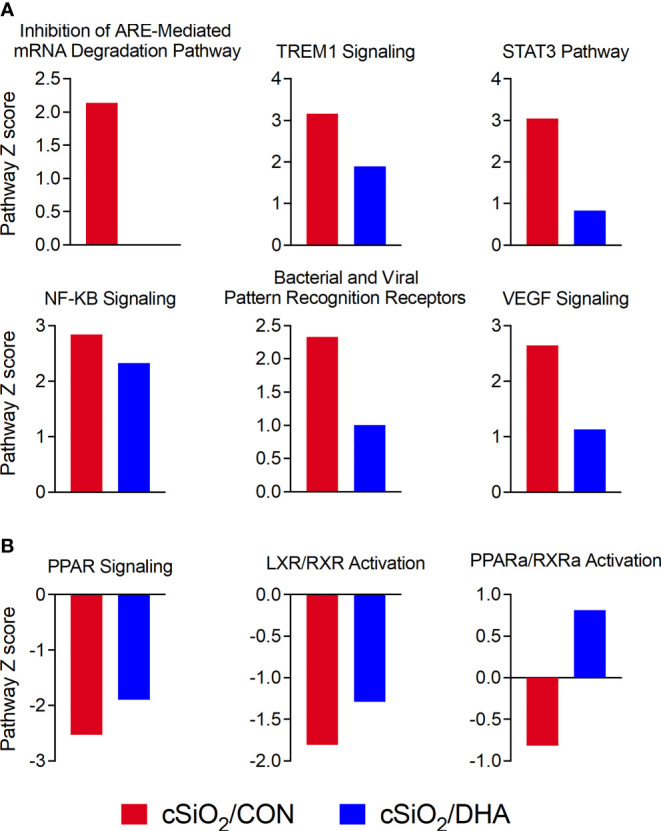
DHA-rich diet suppresses cSiO_2_-triggered immune-related pathways and reverses cSiO_2_-induced suppression of lipid metabolism pathways. Canonical pathway analysis was performed for comparison of inflammatory proteins from cSiO_2_/CON (blue bars) and cSiO_2_/DHA groups (red bars) using IPA tools. Significant (p ≤ 0.05) Z-scores that were greater than 2 (absolute value) for immune-related function **(A)** and lipid metabolism **(B)** annotations are shown as bar graphs.

IPA was further utilized to identify functionally linked pathways that were significantly influenced by cSiO_2_ and DHA (**Figure 10**). DHA’s effects were associated with downregulation of cSiO_2_-induced pathways involving ARE‐mediated mRNA decay, TREM1 signaling, STAT3, NF-κB signaling, bacterial and viral pattern recognition receptor activation, and VEGF signaling (**Figure 10A**) and upregulation of PPAR signaling, LXR/RXR activation, and PPARα/RXRα activation (**Figure 10B**).

### cSiO_2_ and DHA Had Limited Effects on the Inflammatory Proteome in Plasma

In contrast to BALF, cSiO_2_ and DHA had relatively modest effects on expression of most plasma proteins in the targeted array (**Supplementary Figure 4** and **Supplementary File 1**). cSiO_2_-treated CON-fed mice showed increases in 9, 7, and 15 proteins at 5, 9, and 13 wk PI, respectively. DHA again reduced expression of cSiO_2_-induced proteins, as cSiO_2_-treated DHA-fed mice showed elevations in 0, 1, and 2 proteins at 5, 9, and 13 wk PI, respectively (**Supplementary Figure 5A**). The chemokines were the one functional group affected by the treatments. As illustrated in mean Z-score plots, chemokines were upregulated in plasma in CON-fed mice following cSiO_2_ exposure mirroring effects seen in BALF (**Supplementary Figures 5B, C**). This upregulation was markedly suppressed in DHA-fed mice. Furthermore, chemokine protein responses in BALF in individual mice over the four time points modestly correlated with plasma chemokine protein responses (**Supplementary Figure 5D**). Notably, CCL6, I-TAC, BLC, and MCP-5 at 9 wk PI showed a strong positive correlation with plasma chemokines (**Supplementary Figure 5E**). Altogether, inflammatory protein responses to cSiO_2_ and DHA were much more robust in the pulmonary region than the systemic compartment, suggesting that the lung was the primary nexus for inflammation and autoimmunity in this model.

## Discussion

Since inhaled cSiO_2_ particles are cleared slowly from the lung, they persist and drive a cycle in alveolar macrophages involving phagocytosis, phagolysosome permeabilization, and inflammasome activation that culminates in cell death and reentry of cSiO_2_ into the alveolar space (17). Repetition of this cycle evokes chronic unresolved inflammation in the lung and release of self-antigens, leading to eventual loss of immune tolerance (38, 39). In prior studies with lupus-prone NZBWF1 female mice, we discovered that DHA feeding prior to cSiO_2_ instillation suppresses progressive increases over wks 1, 5, 9, and 13 PI in inflammatory/autoimmune gene expression, inflammatory cell recruitment, and autoantibody production (16, 35, 36). For example, in mice fed a control diet without DHA, cSiO_2_ instillation triggered a mixed inflammatory cell infiltrate in the lung (alveolitis composed of monocytes/macrophages, neutrophils and lymphocytes) with semiquantitative histopathology severity scores of minimal (1 out 4) and mild to moderate (2/3 out of 4) at 1/5 and 9/13 wk PI, respectively (16). Likewise, total inflammatory cells in BALF from these cSiO_2_-instilled mice were modestly increased at 1 and 5 wk PI and dramatically increased by 9 and 13 wk PI, as compared to saline vehicle-instilled animals fed the control diet. In contrast, cSiO_2_ mice fed DHA supplemented diet (human daily equivalent 5 g/day) had minimal alveolitis at 1,5, and 9 wk PI and only mild alveolitis at 13 wk PI. Total inflammatory cell numbers in BALF of these DHA-supplemented mice were similarly suppressed at 9 and 13 wk PI (16).

The findings presented herein show for the first time how cSiO_2_ and DHA influence the inflammatory proteome in BALF and plasma in this preclinical model of environment-triggered autoimmunity. First, cSiO_2_ induced a diverse inflammatory proteome in the BALF that persisted and progressed long after particle exposure was ceased. Upregulated proteins included chemokines, enzymes, adhesion molecules, co-stimulatory molecules, growth factors, TNF superfamily members, and signal transduction proteins. Second, the plasma inflammatory protein responses to cSiO_2_ were limited to chemokines and much less robust than observed in the BALF, confirming the lung to be the central site for inflammation and autoimmunity in this model. Third, DHA consumption markedly suppressed cSiO_2_-induced expression of many of these inflammatory proteins in BALF at wks 1, 5, and 9 PI and, furthermore, DHA’s ameliorative actions against some inflammatory proteins appeared to be lost at 13 wk PI. Fourth, correlation analyses confirmed that DHA’s inhibitory effects on cSiO_2_-induced inflammatory protein signatures corresponded with prior measurements of transcriptional responses, ectopic lymphoid structure development, and autoantibody production. Finally, DHA’s ameliorative effects were associated with inhibition of proinflammatory cytokine-driven innate immune pathways and activation of lipid-signaling signaling pathways. Altogether, these new findings support the contention that DHA supplementation might be a promising intervention against cSiO_2_-induced inflammation and autoimmune disease onset and flaring in susceptible individuals.

IPA analysis indicated that IL-1, IL-6, and TNF-α were among the chief regulators for predicted pathways mediating cSiO_2_-triggered inflammatory proteins and that these pathways were downregulated by DHA. cSiO_2_ instillation into the lung is well-known to acutely elicit secretion of proinflammatory cytokines such as IL-1β, IL-1α, IL-6, and TNF-α by direct or indirect action on alveolar macrophages, neutrophils, and epithelial cells (40–43). Therefore, it was surprising that cSiO_2_’s effects on these proinflammatory cytokines in BALF were modest across the time cohorts of this study. Nevertheless, these low responses concurred with low expression of corresponding cytokine mRNAs in the parent study (35). The lack of a robust proinflammatory cytokine responses to prolonged cSiO_2_ exposure compared to that for chemokines and other proteins likely reflects the strict regulation and relatively short half-lives of proinflammatory cytokine mRNAs and proteins in tissues due to their injurious potential (44).

Pathway analysis further revealed that DHA’s quelling effects were associated with attenuation of ARE‐mediated mRNA decay, TREM1, NF-κB, and STAT3 signaling pathways, and potentiation of PPAR signaling and PPARα/RXRα and LXR/RXR activation. In the parent investigation from which tissue fluids were sourced, we reported that total ω-3 PUFA and ω-6 PUFA represented 6.6 and 33.4% of lung lipids in cSiO_2_-treated CON-fed mice, respectively, whereas in the cSiO_2_-treated DHA-fed group, they were 23.2 and 16.3%, respectively (16). At the mechanistic level, increasing the ω-3:ω-6 PUFA ratio might hamper lipid raft development and prevent activation of transmembrane receptors that control innate and adaptive immune processes (45). Additionally, DHA and EPA can be liberated from the membrane by intracellular and extracellular phospholipases (46, 47), allowing them to act as ligands for extracellular and intracellular receptors that inhibit proinflammatory signaling pathways (48, 49). Consistent with our IPA pathway analysis here, these receptors include PPAR and RXR family members that downregulate NF-κB- and STAT-dependent transcription of innate immune genes (50, 51). Moreover, DHA and EPA can directly compete with ω-6 PUFAs as enzymatic substrates for downstream bioactive lipid mediators, leading to reduced proinflammatory metabolites (e.g. prostaglandins, leukotrienes) and increased specialized pro-resolving metabolites (e.g. resolvins, protectins, maresins) that promote resolution of inflammation (52, 53). Accordingly, changing the tissue balance by enhancing ω-3 PUFAs and diminishing ω-6 PUFAs likely reduces the cSiO_2_-induced inflammatory proteome by several independent or interdependent mechanisms.

The diverse array of inflammatory proteins induced by cSiO_2_ in this lupus-prone mouse model provides new insights into how the particle triggers inflammation and autoimmunity and how such triggering might be ameliorated by consumption of DHA. Chemokines were foremost among the proteins induced by cSiO_2_. The robust increases in BLC (B-lymphocyte chemoattractant; CXCL13) are particularly pertinent in ELT neogenesis. BLC, which is produced by follicular dendritic cells, T follicular helper cells, and Th17 cells, is essential for B-cell localization into lymphoid follicles. Other CXCL chemokines might also impact cSiO_2_-induced autoimmunity. MIP-2 (CXCL3) primarily recruits neutrophils to sites of inflammation that, upon degranulation, could secrete proteolytic enzymes that elicit bystander tissue injury and inflammation, or release neutrophil extracellular DNA traps *via* NETosis, a source of self-DNA and autoantigens (54). Consistent with the increase in CD3^+^ T cells in lung ELT observed in the original study (16), MIG (CXCL9) and ITAC (CXCL11) recruit and position activated T-cells (55). CCL chemokines can also recruit monocytes, dendritic cells, lymphocytes, and eosinophils, which collectively promote cSiO_2_-induced inflammation and autoimmunity in pleiotropic manners (56–58).

cSiO_2_-triggered release of extracellular enzymes into the alveolar fluid and suppression of these responses by DHA also have significant implications to inflammation and autoimmunity in the lung. cSiO_2_ induction of matrix metalloproteinases (MMPs), a family of enzymes that degrade extracellular matrix and basement membrane components, is consistent with prior *in vivo* and *in vitro* investigations (59). cSiO_2_ also elicited extracellular granzyme B, which has been linked to lupus pathogenesis by cleaving autoantigens and exposing neoepitopes, as well as ACE and the aspartic acid protease renin 1, two key enzymes in the renin-angiotensin system. The latter regulates many physiological functions, including inflammation, and has been previously shown to play a role in silicosis in mice and rats (60–64).

Elevated soluble adhesion molecules in the BALF following cSiO_2_ instillation are highly indicative of persistent inflammation, extracellular matrix breakdown, and potentially, as a means of negative feedback inhibition in the lung. Selectins have a key role in inflammation by mediating leukocyte rolling, adhesion, and transmigration into inflamed tissues. Leukocyte (L)-selectin (CD62L) is expressed on neutrophils, monocytes, and most lymphocytes (65) and is a biomarker of inflammation and autoimmune disease, and notably, occupational exposure to cSiO_2_ (62–64). Endothelial (E)-selectin (CD62E) and platelet (P)-selectin are biomarkers in autoimmune and rheumatic diseases (66–69). DHA suppression of adhesion molecules in the alveolar fluid observed in this study is remarkably in agreement with the findings in plasma of prior preclinical and clinical investigations of ω-3 PUFAs (70).

Consistent with extracellular matrix breakdown, cSiO_2_ induced release of soluble TNF superfamily members into the alveolar fluid, and their induction was suppressed by DHA consumption. The ligand-receptor pairs CD40L-CD40 and OX40L-OX40 represent critical immune checkpoints for B cell differentiation, antigen presenting cell activation, and T cell modulation (71). High serum levels sCD40L or sOX40L have been reported in lupus and other rheumatic autoimmune diseases (72, 73). CD6, a lymphocyte surface receptor that associates with the T cell receptor/CD3 complex, acts as a co-stimulatory molecule for T cell activation (74). sCD6 is detectable in patients with systemic autoimmune and inflammatory disorders (75, 76). Other TNF receptor superfamily proteins that were upregulated by cSiO_2_, downregulated by DHA, and have pertinence to lupus and autoimmunity include sBAFFR (77), sTNFR1 and sTNFR2 (43), and sTRAIL (78–80).

Strengths of this investigation include employment of a widely utilized lupus-prone mouse model, a well-recognized autoimmune trigger, and a human-relevant dose of DHA. However, this investigation had several limitations that require additional consideration in future studies. Since this was a discovery-based approach, we employed a single standard dilution for BALF and plasma and used MFI as a measure of protein signal, thus limiting our ability to perform absolute quantitation of inflammatory proteins identified herein. Nevertheless, proteins of further interest can be quantified by ELISA in future studies employing our preclinical model. Additionally, the dose of cSiO_2_ employed here to induce autoimmunity in the NZBWF1 mouse represents one half of a human equivalent lifetime exposure to cSiO_2_ at the recommended NIOSH exposure limit (15). The inability to adequately clear particles at this high dose likely unleashes a vicious cycle of inflammation, cell death, and re-release of cSiO_2_. This propensity for unresolved inflammation appeared to overwhelm DHA’s capacity to suppress induction of some inflammatory proteins by wk 13, suggesting there is a limit to its ameliorative action. Thus, additional insights could be gained by replicating this study using lower cSiO_2_ doses and extending experiment duration.

A further limitation of this study is that since we did not include a non-autoimmune strain in our parent investigation, it could be argued that the observed protein signatures may be a generalized inflammatory response to cSiO_2_ that happens to coincide with autoimmunity in this lupus-prone mouse strain. However, in an earlier prior study, we compared the effects to cSiO_2_ and DHA in female NZBWF1 mice to those for one of the parental lines, female NZW/LacJ, which does not exhibit the propensity towards autoimmunity (81). At 12 wk PI, the NZBWF1 mice exhibited marked macrophage, neutrophil, and lymphocyte infiltration with robust ectopic lymphoid tissue neogenesis in the lung that were suppressed by DHA feeding. In comparison, inflammatory cell responses in the lung and kidney to cSiO_2_ were extremely modest in the NZW/LacJ mice but were nonetheless ameliorated by DHA supplementation. Inflammatory and autoimmune responses to cSiO_2_ are likewise much lower in female C57BL/6 mice, another mouse strain with low lupus predilection (15). Thus, it could be speculated that cSiO_2_-induced inflammatory proteome would be similarly less robust in NZW/LacJ and C57Bl/6 mice, however, this supposition will need future confirmation using these non-autoimmune strains and extending the study length beyond three months after cSiO_2_ treatment.

In conclusion, the demonstration here that DHA prevents or delays cSiO_2_-triggered inflammatory protein expression in NZBWF1 mice provides new insights pathways by which ω-3 PUFAs interfere with environmental triggering of chronic inflammation and autoimmune disease. These findings have resonance with one of the four goals of the 2020-2030 Strategic Plan for NIH Nutrition Research which is to develop precision nutrition strategies to reduce the burden of disease in clinical settings (82). The DHA diet used here equates to human intake of 5 g/d, which is a realistic and safe human dose (83). Consistent with our findings, ameliorative effects on inflammatory mediators or inflammatory cell function in human investigations have been found at marine ω-3 PUFA intakes of > 2 g/d (21), and a recent meta-analysis of human clinical trials determined that supplementation with 3 g/d could benefit patients with lupus (33). Taken together, ω-3 supplementation could be a precision nutrition approach for delaying progression of autoimmunity and reducing lupus flaring following respiratory exposures to cSiO_2_ or potentially other airborne particles.

## Data Availability Statement

Original proteomic datasets from this study are available in a publicly accessible repository: https://doi.org/10.26078/W1G0-KB31. Other existing gene and autoantibody expression datasets used in this study are available at https://doi.org/10.26078/4697-1p77 and https://datadryad.org/stash/share/LLav72h-OvhiPYfqma2-qzCSzeZCJjIts55UWwaXqzA, respectively.

## Ethics Statement

The animal study was reviewed and approved by Institutional Animal Care and Use Committee at Michigan State University (AUF #01/15-021-00).

## Author Contributions

LR: data curation, data analysis/interpretation, figure preparation, manuscript writing. MB: study design, animal study, necropsy, immunohistochemical analyses and morphometry, manuscript preparation and manuscript editing. AB and KW: data analysis/interpretation, figure preparation, manuscript editing. JH: study design, lung/kidney histopathology, morphometry, data analyses. JP: study design, coordination, oversight, funding acquisition, manuscript preparation and submission. All authors contributed to the article and approved the submitted version.

## Funding

This research was funded by NIH ES027353 (JP), NIH F31ES030593 (KW), NIH T32ES007255 (KW), Lupus Foundation of America (JP, KW), USDA National Institute of Food and Agriculture Hatch Projects 1020129 (JP) and UTA-01407 and UTA-01456 (AB), and the Dr.Robert and Carol Deibel Family Endowment (JP).

## Conflict of Interest

The authors declare that the research was conducted in the absence of any commercial or financial relationships that could be construed as a potential conflict of interest.

## Publisher’s Note

All claims expressed in this article are solely those of the authors and do not necessarily represent those of their affiliated organizations, or those of the publisher, the editors and the reviewers. Any product that may be evaluated in this article, or claim that may be made by its manufacturer, is not guaranteed or endorsed by the publisher.

## Supplementary Material

Supplementary Figure 1 cSiO_2_-induced cytokines in BALF are modestly decreased by DHA supplementation. **(A)** Heat maps with unsupervised clustering (Euclidian distance method) depict unit variance-scaled mean fluorescent intensity for cytokines measured in the BALF. Blue, red and green in the top bar indicates the VEH/CON, cSiO_2_/CON, and cSiO_2_/DHA groups, respectively, at 1, 5, 9, or 13 wks PI. Scale bar values reflect the range of variance-stabilized mean fluorescent intensities (MFIs), which were centered across rows. **(B)** DHA diet suppresses cSiO_2_-induced selected cytokines in BALF over time. MFIs were obtained using the microarray panel for VEH- or cSiO_2_-exposed mice fed CON or DHA diets. Data for select proteins were analyzed by two-way ANOVA for main effects of timepoint and treatment with *post-hoc* Tukey HSD multiple comparison tests to determine effects of treatment at each time point. Data are mean ± SEM. Main effect p-values for timepoint, treatment, and the interaction of these factors are shown below the plots. Symbols indicate significant differences (p ≤ 0.05) as follows: * for cSiO_2_/CON vs. VEH/CON; # for cSiO_2_/DHA vs. VEH/CON; and + for cSiO_2_/CON vs. cSiO_2_/DHA.

Supplementary Figure 2 cSiO_2_-induced TNF superfamily proteins in BALF are suppressed by intake of DHA-rich diet. Heat maps with unsupervised clustering (Euclidian distance method) depict unit variance-scaled mean fluorescent intensity for TNF superfamily proteins expression measured in the BALF. Blue, red and green in the top bar indicates the VEH/CON, cSiO_2_/CON, and cSiO_2_/DHA groups, respectively, at 1, 5, 9, or 13 wks PI. Scale bar values reflect the range of variance-stabilized MFIs, which were centered across rows. **(B)** DHA diet suppresses selected cSiO_2_-induced TNF superfamily proteins in BALF over time. Mean fluorescent intensiies were obtained using the microarray panel for vehicle (VEH)- or cSiO_2_-exposed mice fed CON or DHA diets. Data for select proteins were analyzed by two-way ANOVA for main effects of timepoint and treatment with *post-hoc* Tukey HSD multiple comparison tests to determine effects of treatment at each time point. Data are mean ± SEM. Main effect p-values for timepoint, treatment, and the interaction of these factors are shown below the plots. Symbols indicate significant differences (p ≤ 0.05) as follows: * for cSiO_2_/CON vs. VEH/CON; # for cSiO_2_/DHA vs. VEH/CON; and +for cSiO_2_/CON vs. cSiO_2_/DHA.

Supplementary Figure 3 cSiO_2_-induced signal transduction proteins expression in BALF are suppressed by intake of DHA rich diet. **(A)** Heat maps with unsupervised clustering (Euclidian distance method) depict unit variance-scaled mean fluorescent intensity for signal transduction proteins expression measured in the BALF. Blue, red and green in the top bar indicates the VEH/CON, cSiO_2_/CON, and cSiO_2_/DHA groups, respectively, at 1, 5, 9, or 13 wks PI. Scale bar values reflect the range of variance-stabilized mean fluorescent intensities (MFIs), which were centered across rows. **(B)** DHA diet suppresses cSiO_2_-induced selected signal transduction proteins in BALF over time. MFIs were obtained using the microarray panel for vehicle (VEH)- or cSiO_2_-exposed mice fed CON or DHA diets. Data for select proteins were analyzed by two-way ANOVA for main effects of timepoint and treatment with *post-hoc* Tukey HSD multiple comparison tests to determine effects of treatment at each time point. Data are mean ± SEM. Main effect p-values for timepoint, treatment, and the interaction of these factors are shown below the plots. Symbols indicate significant differences (p ≤ 0.05) as follows: * for cSiO_2_/CON vs. VEH/CON; # for cSiO_2_/DHA vs. VEH/CON; and +for cSiO_2_/CON vs. cSiO_2_/DHA.

Supplementary Figure 4 Effects of DHA intake on cSiO_2_-induced inflammatory protein responses in plasma. Heat maps with unsupervised clustering (Euclidian distance method) depict mean fluorescent intensities (MFIs) for 203 proteins. Black, red, and Blue in the top bar indicates the VEH/CON, cSiO_2_/CON, and cSiO_2_/DHAgroups, respectively, at 1,5, 9, or 13 wks PI. Scale bar values reflect the range of variance-stabilized mean fluorescent intensities, which were centered across rows.

Supplementary Figure 5 Effects of DHA on cSiO_2_-induced chemokine expression in BALF and plasma are similar. **(A)** Summary of proteins altered in cSiO_2_/CON and cSiO_2_/DHA mice in BALF over time. DHA supplementation suppresses cSiO_2_-induced chemokine expression in plasma **(B)** and BALF **(C)** over time. Z- score for chemokine proteins was calculated using mean fluorescent intensities of chemokine proteins belonging to chemokine category (**Supplemental Table 1**). Missing values and outliers were handled as described in the Methods section. Data for select proteins were analyzed by two-way ANOVA for main effects of timepoint and treatment with *post-hoc* Tukey HSD multiple comparison tests to determine effects of treatment at each time point. Data are mean ± SEM. Main effect p-values for timepoint, treatment, and the interaction of these factors are shown below the plots. Symbols indicate significant differences (p ≤ 0.05) as follows: * for cSiO_2_/CON vs. VEH/CON; # for cSiO_2_/DHA vs. VEH/CON; and +for cSiO_2_/CON vs. cSiO_2_/DHA. **(D)** BALF chemokine protein responses positively correlate with corresponding plasma chemokine protein responses. Scatter plots for Z scores from chemokines in plasma vs. Z scores from chemokine in BALF in each animal. Linear regression lines with 95% confidence intervals (curved lines) are shown along with the Spearman ρ value and p-value. **(E)** Correlation between selected individual chemokines in BALF and same chemokine in plasma for individual animals was assessed by Spearman’s correlation coefficient at wk 9. *p < 0.05.
