# Supplementary material for: Silica Induction of Diverse Inflammatory Proteome in Lungs of Lupus-Prone Mice Quelled by Dietary Docosahexaenoic Acid Supplementation
Source: Front Immunol. 2022 Jan 21;12:781446. doi: 10.3389/fimmu.2021.781446 (PMC8813772; doi:10.3389/fimmu.2021.781446)
Supplement: Supplementary Figure 1 — cSiO2-induced cytokines in BALF are modestly decreased by DHA supplementation. (A) Heat maps with unsupervised clustering (Euclidian distance method) depict unit variance-scaled mean fluorescent intensity for cytokines measured in the BALF. Blue, red and green in the top bar indicates the VEH/CON, cSiO2/CON, and cSiO2/DHA groups, respectively, at 1, 5, 9, or 13 wks PI. Scale bar values reflect the range of variance-stabilized mean fluorescent intensities (MFIs), which were centered across rows. (B) DHA diet suppresses cSiO2-induced selected cytokines in BALF over time. MFIs were obtained using the microarray panel for VEH- or cSiO2-exposed mice fed CON or DHA diets. Data for select proteins were analyzed by two-way ANOVA for main effects of timepoint and treatment with post-hoc Tukey HSD multiple comparison tests to determine effects of treatment at each time point. Data are mean ± SEM. Main effect p-values for timepoint, treatment, and the interaction of these factors are shown below the plots. Symbols indicate significant differences (p ≤ 0.05) as follows: * for cSiO2/CON vs. VEH/CON; # for cSiO2/DHA vs. VEH/CON; and + for cSiO2/CON vs. cSiO2/DHA. [file DataSheet_1.pdf]

Silica Induction of Diverse Inflammatory Proteome in Lungs of Lupus-prone Mice Quelled by Dietary Docosahexaenoic Acid Supplementation

Lichchavi D. Rajasinghe[1,2*], Melissa A. Bates[1,2*] , Abby D Benninghoff [3], Kathryn A. Wierenga[4], Jack R. Harkema[5], and James J. Pestka[1,2,6]**

Supplemental materials

1. **Supplemental Table 1.** Classification of proteins by cellular function.
2. **Supplemental Table 2**. Target molecules of upstream regulators
3. **Supplemental Figure 1**. $cSiO_2$-induced cytokines in BALF are modestly decreased by DHA supplementation.
4. **Supplemental Figure 2**. cSiO2-induced TNF Superfamily proteins in BALF are suppressed by intake of DHA rich diet.
5. **Supplemental Figure 3**. $cSiO_2$-induced signal transduction proteins expression in BALF are suppressed by intake of DHA rich diet.
6. **Supplemental Figure 4**. Effects of DHA intake on $cSiO_2$-induced inflammatory protein responses in plasma.
7. **Supplemental Figure 5.** Effects of DHA on cSiO2-induced chemokine expression in BALF and plasma are similar.

Supplementary Table 1. Classification of proteins by cellular function

Uniprot	Ensembl protein ID	Functional Category	Official Symbol	Target name	Protein names
P09803	ENSMUSG00000000303	Adhesion molecule	Cdh1	E-Cadherin	Cadherin-1
Q9QYY7	ENSMUSP00000040187	Adhesion molecule	Esm1	Endocan	Endothelial cell-specific molecule 1
Q00690	ENSMUSG00000026582	Adhesion molecule	Sele	E-Selectin	E-selectin
P13597	ENSMUSP00000083587	Adhesion molecule	Icam1	ICAM-1	Intercellular adhesion molecule 1
O88792	ENSMUSP00000041907	Adhesion molecule	F11r	JAM-A	Junctional adhesion molecule A
P18337	ENSMUSP00000027871	Adhesion molecule	Sell	L-Selectin	L-selectin
Q61826	ENSMUSP00000151928	Adhesion molecule	Madcam1	MAdCAM-1	Mucosal addressin cell adhesion molecule 1
P10287	ENSMUSP00000079613	Adhesion molecule	Cdh3	P-Cadherin	Cadherin-3
Q62009	ENSMUSP00000072773	Adhesion molecule	Postn	Periostin	Periostin
Q01102	ENSMUSP00000123924	Adhesion molecule	Selp	P-Selectin	P-selectin
P29533	ENSMUSP00000142876	Adhesion molecule	Vcam1	VCAM-1	Vascular cell adhesion protein 1
P86792	ENSMUSP00000095727	Chemokine	Ccl21b	6Ckine	C-C motif chemokine 21b
O55038	ENSMUSP00000023840	Chemokine	Cxcl13	BLC	C-X-C motif chemokine 13
P06684	ENSMUSP00000028233	Chemokine	C5	C5	Complement C5
P10146	ENSMUSP00000103824	Chemokine	Ccl1	CCL1	C-C motif chemokine 1
Q9JIL2	ENSMUSP00000096847	Chemokine	Ccl28	CCL28	C-C motif chemokine 28
P27784	ENSMUSP00000019071	Chemokine	Ccl6	CCL6	C-C motif chemokine 6
Q8BSU2	ENSMUSP00000019064	Chemokine	Cxcl16	CXCL16	C-X-C motif chemokine 16
P50228	ENSMUSP00000031318	Chemokine	Cxcl5	CXCL5	C-X-C motif chemokine 5
P48298	ENSMUSP00000000342	Chemokine	Ccl11	Eotaxin-1	Eotaxin
Q9JKC0	ENSMUSP00000004936	Chemokine	Ccl24	Eotaxin-2	C-C motif chemokine 24
O35188	ENSMUSP00000034230	Chemokine	Cx3cl1	Fractalkine	Fractalkine
Q9JHH5	ENSMUSG00000060183	Chemokine	Cxcl11	I-TAC	C-X-C motif chemokine 11
P12850	ENSMUSP00000031327	Chemokine	Cxcl1	KC	Growth-regulated alpha protein
Q9WVL7	ENSMUSP00000031322	Chemokine	Cxcl15	Lungkine	C-X-C motif chemokine 15
P47993	ENSMUSP00000027860	Chemokine	Xcl1	Lymphotactin	Lymphotactin

Supplementary Table 1. Classification of proteins by cellular function

Uniprot	Ensembl protein ID	Functional Category	Official Symbol	Target name	Protein names
P10148	ENSMUSP00000000193	Chemokine	Ccl2	MCP-1	C-C motif chemokine 2
Q62401	ENSMUSP00000000194	Chemokine	Ccl12	MCP-5	C-C motif chemokine 12
O88430	ENSMUSP00000034231	Chemokine	Ccl22	MDC	C-C motif chemokine 22
P18340	ENSMUSP00000108716	Chemokine	Cxcl9	MIG	C-X-C motif chemokine 9
P10855	ENSMUSP00000001008	Chemokine	Ccl3	MIP-1 alpha	C-C motif chemokine 3
P14097	ENSMUSP00000019074	Chemokine	Ccl4	MIP-1 beta	C-C motif chemokine 4
P51670	ENSMUSP00000019266	Chemokine	Ccl9	MIP-1 gamma	C-C motif chemokine 9
P10889	ENSMUSP00000074885	Chemokine	Cxcl2	MIP-2	C-X-C motif chemokine 2
O89093	ENSMUSP00000109064	Chemokine	Ccl20	MIP-3 alpha	C-C motif chemokine 20
O70460	ENSMUSP00000100022	Chemokine	Ccl19	MIP-3 beta	C-C motif chemokine 19
Q9Z126	ENSMUSP00000031320	Chemokine	Pf4	Platelet Factor 4	Platelet factor 4
P30882	ENSMUSP00000039600	Chemokine	Ccl5	RANTES	C-C motif chemokine 5
O35903	ENSMUSP00000024004	Chemokine	Ccl25	TECK	C-C motif chemokine 25
P41272	ENSMUSP00000032486	Co-stimulatory molecule	Cd27	CD27	CD27 antigen
O55237	ENSMUSP00000019633	Co-stimulatory molecule	Cd70	CD27 Ligand	CD70 antigen
P27548	ENSMUSP00000033466	Co-stimulatory molecule	Cd40lg	CD40 Ligand	CD40 ligand
P18181	ENSMUSP00000064241	Co-stimulatory molecule	Cd48	CD48	CD48 antigen
Q61003	ENSMUSP00000046861	Co-stimulatory molecule	Cd6	CD6	T-cell differentiation antigen CD6
Q00609	ENSMUSP00000097404	Co-stimulatory molecule	Cd80	CD80	T-lymphocyte activation antigen CD80
P09793	ENSG00000163599	Co-stimulatory molecule	Ctla4	CTLA-4	Cytotoxic T-lymphocyte protein 4
P26955	ENSMUSP00000094082	Cytokine	Csf2rb	GM-CSF	Cytokine receptor common subunit beta
Q00560	ENSMUSP00000064205	Cytokine	Il6st	gp130	Interleukin-6 receptor subunit beta
O70326	ENSMUSP00000097170	Cytokine	Grem1	Gremlin-1	Gremlin-1
P01580	ENSMUSP00000063800	Cytokine	Ifng	IFN-gamma	Interferon gamma
P15261	ENSMUSP00000020188	Cytokine	Ifngr1	IFN-gamma R1	Interferon gamma receptor 1
P01582	ENSMUSP00000028882	Cytokine	Il1a	IL-1 alpha	Interleukin-1 alpha

Supplementary Table 1. Classification of proteins by cellular function

Uniprot	Ensembl protein ID	Functional Category	Official Symbol	Target name	Protein names
P10749	ENSMUSP00000028881	Cytokine	Il1b	IL-1 beta	Interleukin-1 beta
P14719	ENSMUSP00000054914	Cytokine	Il1rl1	IL-1 R4	Interleukin-1 receptor-like 1
P25085	ENSMUSP00000110126	Cytokine	Il1rn	IL-1 Ra	Interleukin-1 receptor antagonist protein
P18893	ENSMUSP00000016673	Cytokine	Il10	IL-10	Interleukin-10
P43432	ENSMUSP00000099860	Cytokine	Il12b	IL-12 p40	Interleukin-12 subunit beta
P43431	ENSMUSP00000103446	Cytokine	Il12a	IL-12 p70	Interleukin-12 subunit alpha
P20109	ENSMUSP00000020650	Cytokine	Il13	IL-13	Interleukin-13
P48346	ENSMUSP00000034148	Cytokine	Il15	IL-15	Interleukin-15
Q9JIP3	ENSMUSP00000016110	Cytokine	Il17rb	IL-17 RB	Interleukin-17 receptor B
Q62386	ENSMUSP00000027061	Cytokine	Il17a	IL-17A	Interleukin-17A
Q9QXT6	ENSMUSP00000025471	Cytokine	Il17b	IL-17B	Interleukin-17B
Q7TNI7	ENSMUSP00000046960	Cytokine	Il17f	IL-17F	Interleukin-17F
P04351	ENSMUSP00000029275	Cytokine	Il2	IL-2	Interleukin-2
P01590	ENSMUSP00000028111	Cytokine	Il2ra	IL-2 R alpha	Interleukin-2 receptor subunit alpha
Q9JKV9	ENSMUSP00000027673	Cytokine	Il20	IL-20	Interleukin-20
Q9ES17	ENSMUSP00000029273	Cytokine	Il21	IL-21	Interleukin-21
Q9JJY9	ENSMUSP00000094449	Cytokine	Il22	IL-22	Interleukin-22
Q9EQ14	ENSMUSP00000026449	Cytokine	Il23a	IL-23 p19	Interleukin-23 subunit alpha
Q4VK74	ENSMUSP00000080384	Cytokine	Ifnl2	IL-28A	Interferon lambda-2
P01586	ENSMUSP00000019058	Cytokine	Il3	IL-3	Interleukin-3
P26954	ENSMUSP00000094083	Cytokine	Csf2rb2	IL-3 R beta	Interleukin-3 receptor class 2 subunit beta
Q8BVZ5	ENSMUSP00000025724	Cytokine	Il33	IL-33	Interleukin-33
P07750	ENSMUSP00000000889	Cytokine	Il4	IL-4	Interleukin-4
P04401	ENSMUSP00000043369	Cytokine	Il5	IL-5	Interleukin-5
P08505	ENSMUSP00000026845	Cytokine	Il6	IL-6	Interleukin-6
P10168	ENSMUSP00000141845	Cytokine	Il7	IL-7	Interleukin-7

Supplementary Table 1. Classification of proteins by cellular function

Uniprot	Ensembl protein ID	Functional Category	Official Symbol	Target name	Protein names
P16872	ENSMUSP00000003981	Cytokine	Il7r	IL-7 R alpha	Interleukin-7 receptor subunit alpha
P15247	ENSMUSP00000022019	Cytokine	Il9	IL-9	Interleukin-9
Q61716	ENSMUSP00000127921	Cytokine	Ifna11	Limitin	Interferon alpha-11
P10923	ENSMUSP00000031243	Cytokine	Spp1	Osteopontin	Osteopontin
Q9JIE6	ENSMUSP00000025237	Cytokine	Tslp	TSLP	Thymic stromal lymphopoietin
P09470	ENSMUSP00000095727	Enzyme	Ace	ACE	Angiotensin-converting enzyme
P97857	ENSMUSP00000023610	Enzyme	Adamts1	ADAMTS-1	A disintegrin and metalloproteinase with thrombospondin motifs 1
Q61288	ENSMUSP00000000542	Enzyme	Acvrl1	ALK-1	Serine/threonine-protein kinase receptor R3
Q00993	ENSMUSP00000002677	Enzyme	Axl	AXL	Tyrosine-protein kinase receptor UFO
Q8VDG3	ENSMUSG00000022685	Enzyme	Parn	DAN	Poly-specific ribonuclease PARN
P55144	ENSMUSP00000028763	Enzyme	Tyro3	Dtk	Tyrosine-protein kinase receptor TYRO3
P49772	ENSMUSP00000123506	Enzyme	Flt3lg	Flt-3 Ligand	Fms-related tyrosine kinase 3 ligand
P04187	ENSMUSP00000015581	Enzyme	Gzmb	Granzyme B	Granzyme B
Q8BJR6	ENSMUSP00000056483	Enzyme	Prss27	Marapsin	Serine protease 27
O55123	ENSMUSP00000034488	Enzyme	Mmp10	MMP-10	Stromelysin-2
P33434	ENSMUSP00000034187	Enzyme	Mmp2	MMP-2	72 kDa type IV collagenase
P28862	ENSMUSG00000043613	Enzyme	Mmp3	MMP-3	Stromelysin-1
Q61391	ENSMUSP00000029400	Enzyme	Mme	Neprilysin	Neprilysin
P41245	ENSMUSP00000017881	Enzyme	Mmp9	Pro-MMP-9	Matrix metalloproteinase-9
Q9ESD1	ENSMUSG00000030800	Enzyme	Prss8	Prostasin	Prostasin
Q9WVS4	ENSMUSP00000068904	Enzyme	Mok	RAGE	MAPK/MAK/MRK overlapping kinase
P06281	ENSMUSP00000092135	Enzyme	Ren1	Renin 1	Renin-1
P20826	ENSMUSP00000020129	Enzyme	Kitlg	SCF	Kit ligand
Q9ER10	ENSMUSG00000013663	Enzyme	Prss22	Tryptase epsilon	Brain-specific serine protease 4
P21460	ENSMUSP00000028938	Enzyme	Cst3	Cystatin C	Cystatin-C

Supplementary Table 1. Classification of proteins by cellular function

Uniprot	Ensembl protein ID	Functional Category	Official Symbol	Target name	Protein names
Q04998	ENSMUSP00000047894	Growth factor	Inhba	Activin A	Inhibin beta A chain
Q9Z0L2	ENSMUSP00000064521	Growth factor	Artn	Artemin	Artemin
Q05928	ENSMUSP00000112765	Growth factor	Btc	Betacellulin	Probetacellulin
P15655	ENSMUSP00000122227	Growth factor	Fgf2	bFGF	Fibroblast growth factor 2
Q60753	ENSMUSP00000049161	Growth factor	Ctf1	Cardiotrophin-1	Cardiotrophin-1
Q9Z0E2	ENSMUSP00000007171	Growth factor	Chrd	Chordin	Chordin
P28654	ENSMUSP00000100924	Growth factor	Dcn	Decorin	Decorin
O54908	ENSMUSP00000025803	Growth factor	Dkk1	DKK-1	Dickkopf-related protein 1
P01132	ENSMUSP00000029653	Growth factor	Egf	EGF	Pro-epidermal growth factor
Q63961	ENSMUSP00000009705	Growth factor	Eng	Endoglin	Endoglin
Q924X1	ENSMUSP00000046987	Growth factor	Epgn	Epigen	Epigen
Q61521	ENSMUSP00000031324	Growth factor	Ereg	Epiregulin	Proepiregulin
P29699	ENSMUSP00000023583	Growth factor	Ahsg	Fetuin A	Alpha-2-HS-glycoprotein
Q01721	ENSMUSG00000052957	Growth factor	Gas1	Gas 1	Growth arrest-specific protein 1
Q61592	ENSMUSP00000033828	Growth factor	Gas6	Gas 6	Growth arrest-specific protein 6
P09920	ENSMUSP00000037762	Growth factor	Csf3	GCSF	Granulocyte colony-stimulating factor
Q9R097	ENSMUSP00000028783	Growth factor	Spint1	HAI-1	Kunitz-type protease inhibitor 1
Q08048	ENSMUSP00000030683	Growth factor	Hgf	HGF	Hepatocyte growth factor
P16056	ENSMUSG00000009376	Growth factor	Met	HGFR	Hepatocyte growth factor receptor
P05017	ENSMUSP00000093005	Growth factor	Igf1	IGF-1	Insulin-like growth factor I
P47877	ENSMUSP00000046610	Growth factor	Igfbp2	IGFBP-2	Insulin-like growth factor-binding protein 2
P47878	ENSMUSP00000020702	Growth factor	Igfbp3	IGFBP-3	Insulin-like growth factor-binding protein 3
Q07079	ENSMUSP00000027377	Growth factor	Igfbp5	IGFBP-5	Insulin-like growth factor-binding protein 5
P47880	ENSMUSP00000023807	Growth factor	Igfbp6	IGFBP-6	Insulin-like growth factor-binding protein 6
P07141	ENSMUSP00000014743	Growth factor	Csf1	M-CSF	Macrophage colony-stimulating factor 1
Q8C1Q4	ENSMUSP00000002344	Growth factor	Metrn	Meteorin	Meteorin

Supplementary Table 1. Classification of proteins by cellular function

Uniprot	Ensembl protein ID	Functional Category	Official Symbol	Target name	Protein names
P20033	ENSMUSG00000025856	Growth factor	Pdgfa	PDGF-AA	Platelet-derived growth factor subunit A
O70300	ENSMUSG00000002664	Growth factor	Pspn	Persephin	Persephin
P49764	ENSMUSP00000004913	Growth factor	Pgf	PLGF-2	Placenta growth factor
P28798	ENSMUSG00000034708	Growth factor	Grn	Progranulin	Granulins
P06879	ENSMUSG00000079092	Growth factor	Prl	Prolactin	Prolactin
P40224	ENSMUSP00000108487	Growth factor	Cxcl12	SDF-1 alpha	Stromal cell-derived factor 1
Q62226	ENSMUSP00000002708	Growth factor	Shh	Sonic Hedgehog N-Terminal	Sonic hedgehog protein
P04202	ENSMUSP00000002678	Growth factor	Tgfb1	TGF beta 1	Transforming growth factor beta-1
P40226	ENSMUSP00000075756	Growth factor	Thpo	Thrombopoietin	Thrombopoietin
Q00731	ENSMUSP00000024747	Growth factor	Vegfa	VEGF-A	Vascular endothelial growth factor A
P49766	ENSMUSP00000025914	Growth factor	Vegfb	VEGF-B	Vascular endothelial growth factor B
P97946	ENSMUSP00000033751	Growth factor	Vegfd	VEGF-D	Vascular endothelial growth factor D
P35969	ENSMUSG00000029648	Growth factor	Flt1	VEGFR1	Vascular endothelial growth factor receptor 1
P35918	ENSMUSG00000062960	Growth factor	Kdr	VEGFR2	Vascular endothelial growth factor receptor 2
P35917	ENSMUSP00000020617	Growth factor	Flt4	VEGFR3	Vascular endothelial growth factor receptor 3
Q60994	ENSMUSP00000023593	Signal transduction	Adipoq	Adiponectin	Adiponectin
P31955	ENSMUSP00000031325	Signal transduction	Areg	Amphiregulin	Amphiregulin
Q640P2	ENSMUSP00000027885	Signal transduction	Angptl1	ANGPTL1	Angiopoietin-related protein 1
Q9R182	ENSMUSP00000030280	Signal transduction	Angptl3	ANGPTL3	Angiopoietin-related protein 3
Q08857	ENSMUSP00000080974	Signal transduction	Cd36	CD36	Platelet glycoprotein 4
Q9DD06	ENSMUSP00000144793	Signal transduction	Rarres2	Chemerin	Retinoic acid receptor responder protein 2
Q06890	ENSMUSP00000022616	Signal transduction	Clu	Clusterin	Clusterin
P14847	ENSMUSP00000044665	Signal transduction	Crp	CRP	C-reactive protein
Q9JI71	ENSMUSP00000099575	Signal transduction	Dll4	DLL4	Delta-like protein 4
P08101	ENSMUSG00000026656	Signal transduction	Fcgr2	Fc gamma RII	Low affinity immunoglobulin gamma Fc region receptor II

Supplementary Table 1. Classification of proteins by cellular function

Uniprot	Ensembl protein ID	Functional Category	Official Symbol	Target name	Protein names
P16045	ENSMUSP00000086795	Signal transduction	Lgals1	Galectin-1	Galectin-1
P16110	ENSMUSG00000050335	Signal transduction	Lgals3	Galectin-3	Galectin-3
O54974	ENSMUSG00000053522	Signal transduction	Lgals7	Galectin-7	Galectin-7
Q3TDZ7		Signal transduction	H60a	H60	Histocompatibility antigen 60a
Q99N43	ENSMUSP00000020662	Signal transduction	Kremen1	Kremen-1	Kremen protein 1
P41160	ENSMUSP00000067046	Signal transduction	Lep	Leptin	Leptin
P48356	ENSMUSP00000037385	Signal transduction	Lepr	Leptin R	Leptin receptor
P11672	ENSMUSP00000053962	Signal transduction	Lcn2	Lipocalin-2	Neutrophil gelatinase-associated lipocalin
Q9EQ09	ENSMUSP00000032265	Signal transduction	Olr1	LOX-1	Oxidized low-density lipoprotein receptor 1
P41317	ENSMUSP00000025797	Signal transduction	Mbl2	MBL-2	Mannose-binding protein C
P21956	ENSMUSP00000032825	Signal transduction	Mfge8	MFG-E8	Lactadherin
Q9EQS9	ENSMUSP00000045387	Signal transduction	Igdcc4	Nope	Immunoglobulin superfamily DCC subclass member 4
Q64299	ENSMUSP00000054389	Signal transduction	Ccn3	NOV	Protein NOV homolog
Q99P91	ENSMUSP00000031840	Signal transduction	Gpnmb	Osteoactivin	Transmembrane glycoprotein NMB
Q99P87	ENSMUSP00000012849	Signal transduction	Retn	Resistin	Resistin
P97401	ENSMUSP00000028389	Signal transduction	Frzb	sFRP-3	Secreted frizzled-related protein 3
Q9QUM4	ENSMUSP00000015460	Signal transduction	Slamf1	SLAM	Signaling lymphocytic activation molecule
Q8BKV0	ENSMUSP00000091192	Signal transduction	Spock3	Testican 3	Testican-3
Q5QNS5	ENSMUSG00000040405	Signal transduction	Havcr1	TIM-1	Hepatitis A virus cellular receptor 1 homolog
Q9JKE2	ENSMUSP00000038636	Signal transduction	Trem1	TREM-1	Triggering receptor expressed on myeloid cells 1
Q8K558	ENSMUSP00000024792	Signal transduction	Treml1	TREML1	Trem-like transcript 1 protein
P20334	ENSMUSP00000030808	TNF superfamily	Tnfrsf9	4-1BB	Tumor necrosis factor receptor superfamily member 9
Q9D8D0	ENSMUSP00000086564	TNF superfamily	Tnfrsf13c	BAFF R	Tumor necrosis factor receptor superfamily member 13C
Q60846	ENSMUSP00000030339	TNF superfamily	Tnfrsf8	CD30	Tumor necrosis factor receptor superfamily member 8

Supplementary Table 1. Classification of proteins by cellular function

Uniprot	Ensembl protein ID	Functional Category	Official Symbol	Target name	Protein names
P32972	ENSMUSP00000030047	TNF superfamily	Tnfsf8	CD30 Ligand	Tumor necrosis factor ligand superfamily member 8
P27512	ENSMUSP00000017799	TNF superfamily	Cd40	CD40	Tumor necrosis factor receptor superfamily member 5
Q9R187	ENSMUSP00000003312	TNF superfamily	Edar	EDAR	Tumor necrosis factor receptor superfamily member EDAR
P25446	ENSMUSP00000025691	TNF superfamily	Fas	Fas	Tumor necrosis factor receptor superfamily member 6
P41047	ENSMUSP00000000834	TNF superfamily	Faslg	Fas Ligand	Tumor necrosis factor ligand superfamily member 6
O35714	ENSMUSP00000040035	TNF superfamily	Tnfrsf18	GITR	Tumor necrosis factor receptor superfamily member 18
Q7TS55	ENSMUSP00000083251	TNF superfamily	Tnfsf18	GITR Ligand	Tumor necrosis factor ligand superfamily member 18
O08712	ENSMUSP00000078705	TNF superfamily	Tnfrsf11b	Osteoprotegerin	Tumor necrosis factor receptor superfamily member 11B
P43488	ENSMUSP00000028024	TNF superfamily	Tnfsf4	OX40 Ligand	Tumor necrosis factor ligand superfamily member 4
P48759	ENSMUSP00000029421	TNF superfamily	Ptx3	Pentraxin-3	Pentraxin-related protein PTX3
Q9ET35	ENSMUSP00000010286	TNF superfamily	Tnfrsf13b	TACI	Tumor necrosis factor receptor superfamily member 13B
P06804	ENSMUSP00000025263	TNF superfamily	Tnf	TNF alpha	Tumor necrosis factor
P25118	ENSMUSP00000032491	TNF superfamily	Tnfrsf1a	TNF RI	Tumor necrosis factor receptor superfamily member 1A
P25119	ENSMUSP00000030336	TNF superfamily	Tnfrsf1b	TNF RII	Tumor necrosis factor receptor superfamily member 1B
P50592	ENSMUSP00000040271	TNF superfamily	Tnfsf10	TRAIL	Tumor necrosis factor ligand superfamily member 10
O35235	ENSMUSP00000022592	TNF superfamily	Tnfsf11	TRANCE	Tumor necrosis factor ligand superfamily member 11
Q9JLL3	ENSMUSP00000106865	TNF superfamily	Tnfrsf19	TROY	Tumor necrosis factor receptor superfamily member 19
O54907	ENSMUSP00000137972	TNF superfamily	Tnfsf12	TWEAK	Tumor necrosis factor ligand superfamily member 12
Q9CR75	ENSMUSP00000024698	TNF superfamily	Tnfrsf12a	TWEAK R	Tumor necrosis factor receptor superfamily member 12A

Supplementary Table 2. Target molecules of upstream regulators

Upstream Regulator	Molecule Type	Target Molecules of Upstream Regulators
**TNF**	Cytokine	Ace,Adipoq,Ar,C5,Ccl11,Ccl19,Ccl2,Ccl2,Ccl20,Ccl24,Ccl28,Ccl3l3,Ccl4,Ccl5,Ccl6,Ccl9,Cd36,Cd40,Cd40lg,Cd70,Cdh1,Cdh3,Clu,Crp,Csf1,Csf2rb,Csf3,Ctla4,Cx3cl1,Cxcl11,Cxcl12,Cxcl13,Cxcl15,Cxcl16,Cxcl2,Cxcl3,Cxcl6,Cxcl9,Dcn,Dkk1,Dll4,Edar,Eng,Ereg,Esm1,F11r,Fas,Faslg,Fcgr2b,Flt1,Flt4,Frzb,Grn,Hgf,Icam1,Ifng,Ifngr1,Igf1,Igfbp2,Igfbp5,Igfbp6,Il10,Il12a,Il12b,Il13,Il15,Il17a,Il17rb,Il1a,Il1b,Il1rl1,Il1rn,Il2,Il20,Il21,Il22,Il23a,Il25,Il2ra,Il3,Il33,Il4,Il5,Il6,Il7,Il7r,Il9,Inhba,Kdr,Kitlg,Lcn2,Lep,Lgals3,Madcam1,Mbl2,Met,Mmp10,Mmp2,Mmp3,Mmp9,Olr1,Pdgfa,Postn,Ptx3,Rarres2,Retn,Sele,Selp,Shh,Spp1,Tgfb1,Tnf,Tnfrsf11b,Tnfrsf18,Tnfrsf1a,Tnfrsf1b,Tnfrsf9,Tnfsf10,Tnfsf11,Tnfsf8,Trem1,Tslp,Vcam1,Vegfa,Vegfd
**IL1B**	Cytokine	Adamts1,Ccl11,Ccl2,Ccl2,Ccl20,Ccl24,Ccl28,Ccl3l3,Ccl4,Ccl5,Ccl9,Cd40,Cd40lg,Crp,Csf1,Csf2rb,Csf3,Cx3cl1,Cxcl11,Cxcl12,Cxcl13,Cxcl2,Cxcl3,Cxcl6,Cxcl9,Dcn,Dll4,Eng,Fas,Faslg,Fcgr2b,Flt1,Grn,Gzmb,Hgf,Icam1,Ifng,Ifngr1,Igf1,Igfbp3,Igfbp6,Il10,Il12a,Il12b,Il15,Il17a,Il17f,Il1a,Il1b,Il1rl1,Il1rn,Il2,Il20,Il21,Il22,Il23a,Il25,Il2ra,Il3,Il33,Il6,Il7,Il9,Inhba,Kdr,Lcn2,Lep,Mmp10,Mmp2,Mmp3,Mmp9,Olr1,Postn,Ptx3,Ren,Sele,Shh,Slamf1,Spp1,Tgfb1,Tnf,Tnfrsf11b,Tnfrsf1b,Tnfrsf9,Tnfsf10,Tnfsf11,Trem1,Tslp,Vcam1,Vegfa
**IL10**	Cytokine	Ccl11,Ccl19,Ccl2,Ccl2,Ccl20,Ccl3l3,Ccl4,Ccl5,Ccl6,Cd40,Csf1,Csf2rb,Csf3,Ctla4,Cxcl12,Cxcl13,Cxcl2,Cxcl3,Cxcl6,Cxcl9,Dcn,Fas,Faslg,Fcgr2b,Icam1,Ifng,Il10,Il12a,Il12b,Il13,Il17a,Il17f,Il17rb,Il1a,Il1b,Il1rn,Il2,Il21,Il22,Il23a,Il33,Il4,Il5,Il6,Il6st,Il7,Il7r,Il9,Kitlg,Lgals1,Mmp2,Mmp9,Ptx3,Sele,Sell,Slamf1,Tgfb1,Thpo,Tnf,Tnfrsf13b,Tnfrsf13c,Tnfrsf1a,Tnfrsf1b,Tnfsf10,Tnfsf11,Trem1,Vcam1,Vegfa,Xcl1
**IL6**	Cytokine	Acvrl1,Adamts1,Areg,Btc,Ccl11,Ccl2,Ccl2,Ccl20,Ccl24,Ccl3l3,Ccl4,Ccl5,Cd36,Cd40,Cd48,Cdh1,Clu,Crp,Csf1,Csf2rb,Cst3,Cxcl13,Cxcl2,Cxcl3,Cxcl6,Dcn,Ereg,Fas,Faslg,Grem1,Gzmb,Hgf,Icam1,Ifng,Igf1,Igfbp3,Igfbp6,Il10,Il12a,Il12b,Il13,Il15,Il17a,Il17f,Il1rl1,Il1rn,Il2,Il20,Il21,Il22,Il23a,Il25,Il4,Il5,Il6,Il6st,Il7,Il7r,Il9,Kdr,Lcn2,Lep,Lgals1,Met,Mmp10,Mmp2,Mmp3,Mmp9,Pdgfa,Pgf,Shh,Spp1,Tgfb1,Thpo,Tnf,Tnfrsf11b,Tnfrsf12a,Tnfrsf1a,Tnfrsf1b,Tnfsf10,Tnfsf11,Vcam1,Vegfa
**IFNG**	Cytokine	Ace,Adipoq,Areg,Ccl11,Ccl19,Ccl2,Ccl2,Ccl20,Ccl25,Ccl28,Ccl3l3,Ccl4,Ccl5,Ccl6,Ccl9,Cd36,Cd40,Cd40lg,Cdh1,Csf1,Csf2rb,Csf3,Cx3cl1,Cxcl11,Cxcl12,Cxcl16,Cxcl2,Cxcl3,Cxcl6,Cxcl9,Dkk1,Egf,Esm1,F11r,Fas,Faslg,Fcgr2b,Flt1,Flt4,Icam1,Ifng,Ifngr1,Igf1,Il10,Il12a,Il12b,Il13,Il15,Il17a,Il17rb,Il1a,Il1b,Il1rl1,Il1rn,Il2,Il21,Il23a,Il25,Il2ra,Il3,Il4,Il5,Il6,Il7,Il7r,Il9,Inhba,Kdr,Kitlg,Lcn2,Lep,Lgals3,Mmp10,Mmp2,Mmp3,Mmp9,Pdgfa,Pf4,Prl,Prss8,Ptx3,Sele,Sell,Selp,Shh,Slamf1,Spp1,Tgfb1,Thpo,Tnf,Tnfrsf11b,Tnfrsf12a,Tnfrsf1a,Tnfrsf1b,Tnfsf10,Tnfsf11,Tnfsf12,Trem1,Vcam1,Vegfa,Xcl1
**IL17A**	Cytokine	Areg,Btc,Ccl11,Ccl2,Ccl2,Ccl20,Ccl4,Ccl5,Cd40,Cdh1,Crp,Csf3,Cx3cl1,Cxcl12,Cxcl13,Cxcl2,Cxcl3,Cxcl6,Ereg,Fas,Icam1,Ifng,Il10,Il12a,Il13,Il17a,Il17f,Il1a,Il1b,Il1rn,Il20,Il22,Il23a,Il25,Il33,Il4,Il5,Il6,Il9,Lcn2,Lep,Mmp2,Mmp3,Mmp9,Pgf,Sele,Selp,Tnf,Tnfrsf11b,Tnfrsf1b,Tnfsf11,Tnfsf12,Tslp,Vcam1,Vegfa,Vegfd
**IL4**	Cytokine	Ccl11,Ccl2,Ccl2,Ccl20,Ccl24,Ccl25,Ccl3l3,Ccl4,Ccl5,Ccl6,Cd36,Cd40,Cd40lg,Cd6,Csf1,Csf2rb,Csf3,Cx3cl1,Cxcl16,Cxcl2,Cxcl3,Cxcl6,Cxcl9,Fas,Faslg,Fcgr2b,Flt1,Flt3lg,Gzmb,Hgf,Icam1,Ifng,Igf1,Il10,Il12a,Il12b,Il13,Il15,Il17a,Il17f,Il17rb,Il1a,Il1b,Il1rl1,Il1rn,Il2,Il21,Il2ra,Il3,Il33,Il4,Il5,Il6,Il6st,Il7r,Il9,Kitlg,Lgals1,Lgals3,Mmp10,Mmp2,Mmp3,Mmp9,Postn,Prss8,Sele,Sell,Selp,Spp1,Tgfb1,Tnf,Tnfrsf11b,Tnfrsf8,Tnfrsf9,Tnfsf11,Tnfsf18,Tnfsf4,Tnfsf8,Tslp,Tyro3,Vcam1,Vegfa,Xcl1
**NFkB (complex)**	complex	Ccl11,Ccl2,Ccl2,Ccl20,Ccl3l3,Ccl4,Ccl5,Cd36,Cd40,Cd40lg,Cdh1,Clu,Crp,Csf1,Csf3,Cx3cl1,Cxcl12,Cxcl2,Cxcl3,Cxcl6,Cxcl9,Fas,Faslg,Icam1,Ifna4,Ifng,Igfbp2,Il10,Il12a,Il12b,Il13,Il15,Il17a,Il1a,Il1b,Il1rn,Il2,Il22,Il23a,Il2ra,Il4,Il5,Il6,Il7r,Il9,Kdr,Lcn2,Lgals7/Lgals7b,Madcam1,Mbl2,Mmp2,Mmp3,Mmp9,Olr1,Ptx3,Sele,Shh,Spp1,Tgfb1,Tnf,Tnfrsf1a,Tnfrsf9,Tnfsf10,Tnfsf11,Tnfsf4,Tslp,Vcam1,Vegfa

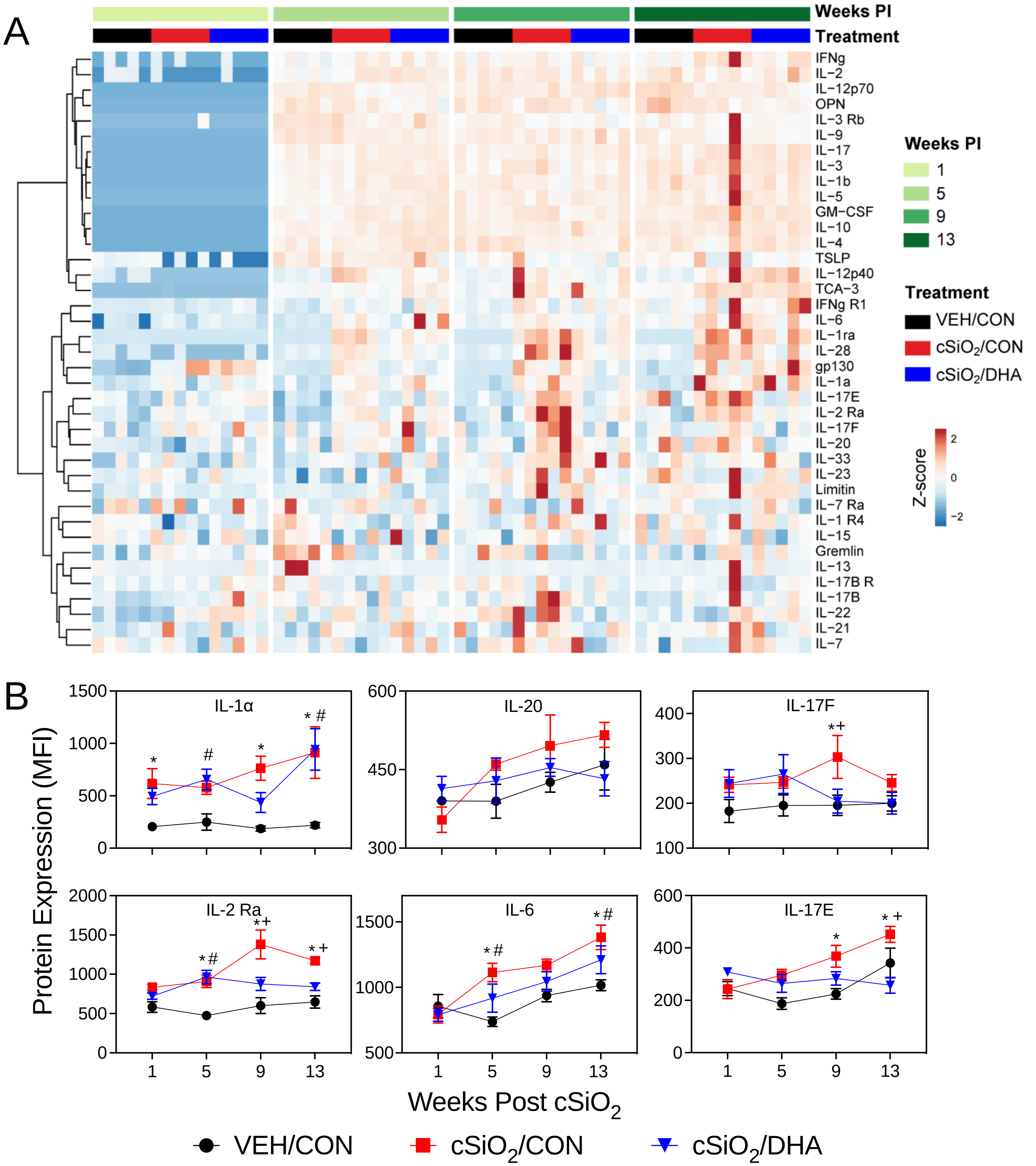

Two-way ANOVA main effect p-values

Factor	Il-1α	IL-20	IL-17F	IL-2 Ra	IL-6	IL-17E
Interaction	0.2987	0.3448	0.4003	0.0102	0.0553	0.0056
Time	0.0483	0.0115	0.7670	0.0045	<0.0001	0.0017
Treatment	<0.0001	0.2092	0.0062	<0.0001	0.0001	0.0008

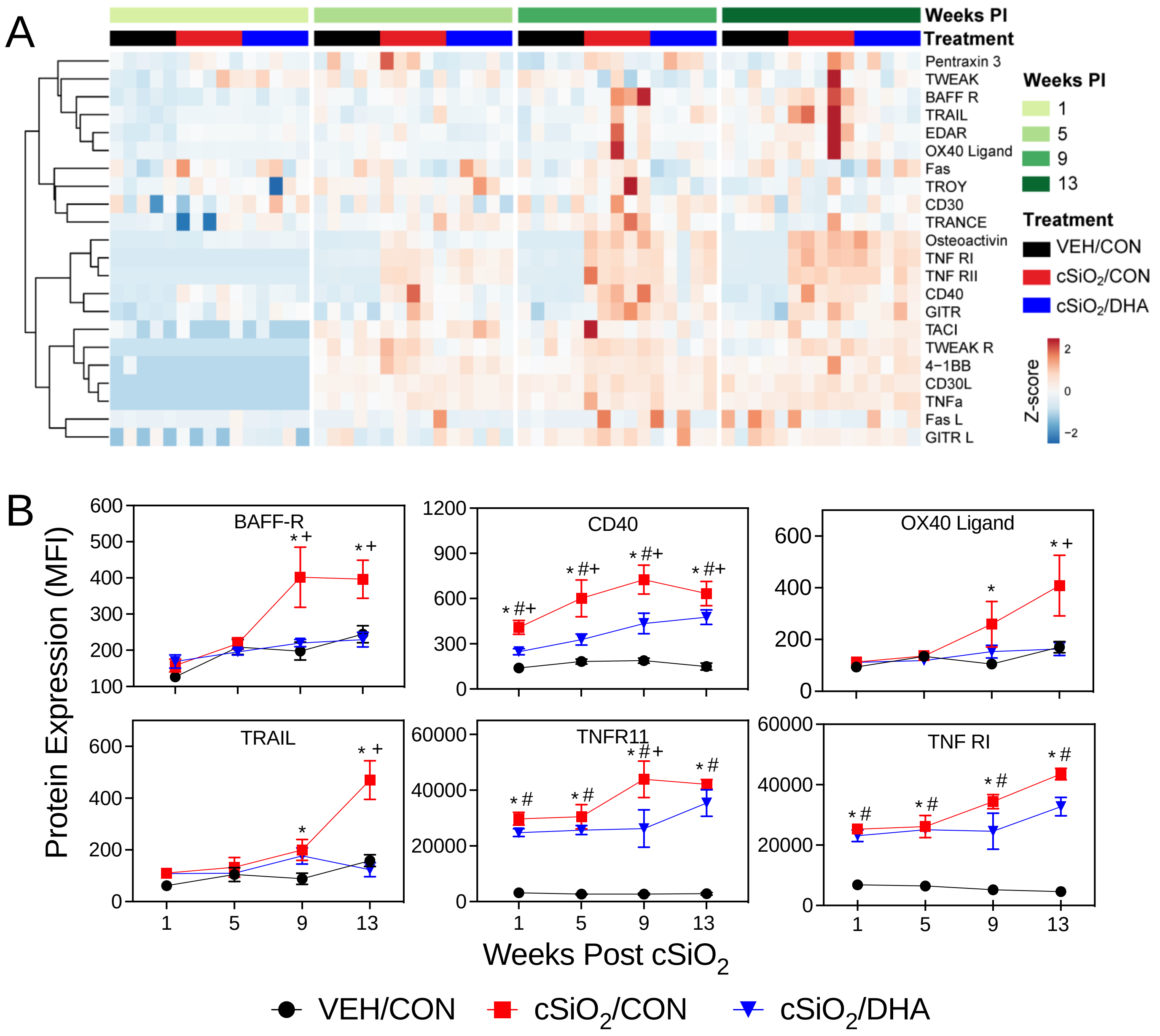

Two-way ANOVA main effect p-values

Factor	BAFF-R	CD40	OX40 Ligand	TRAIL	TNFR11	TNF RI
Interaction	0.0141	0.2603	0.0618	<0.0001	0.1424	0.0056
Time	<0.0001	0.0024	0.0018	<0.0001	0.0244	0.0012
Treatment	<0.0001	<0.0001	0.0032	<0.0001	<0.0001	<0.0001

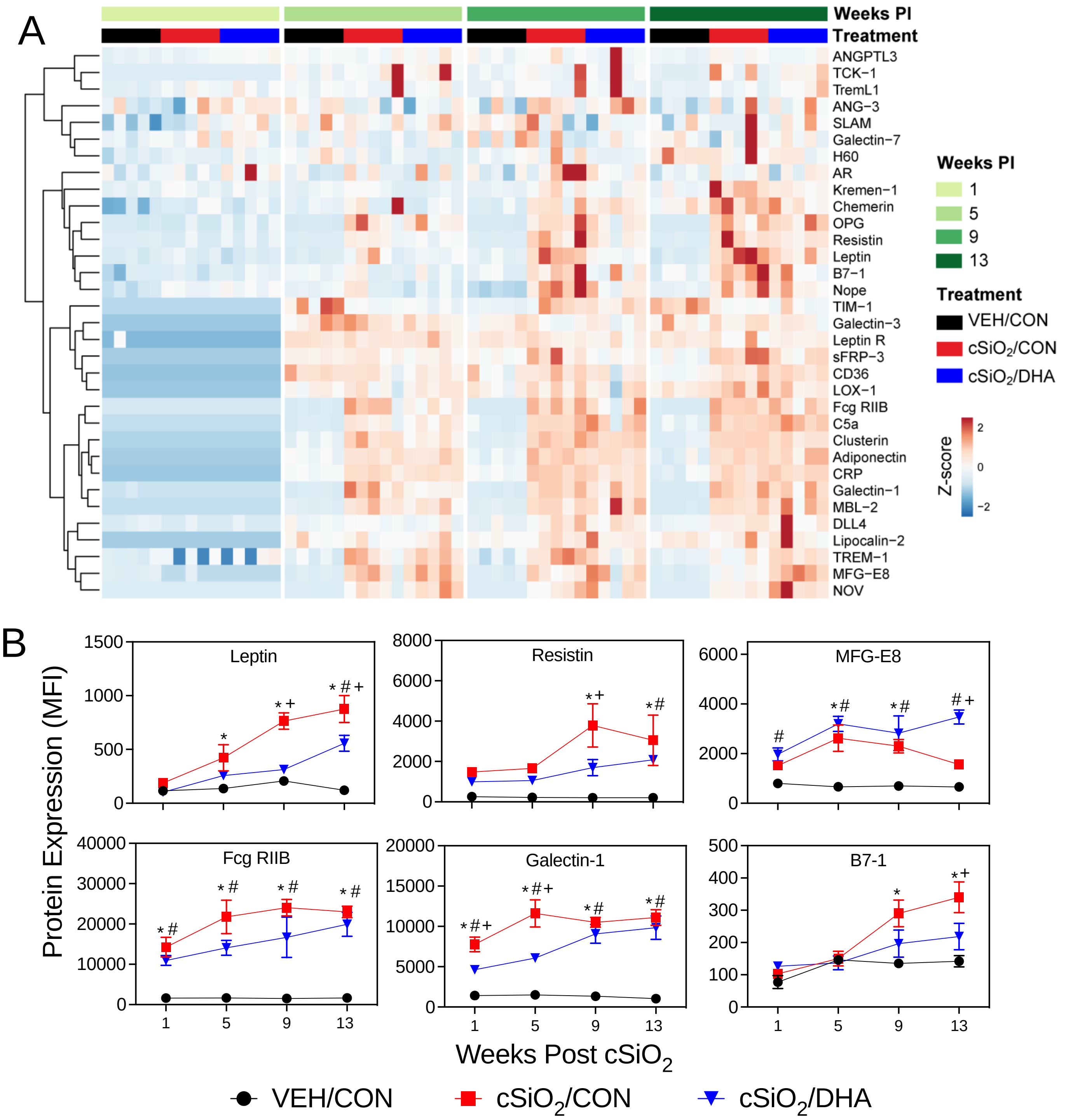

Two-way ANOVA main effect p-values

Factor	Leptin	Resistin	MFG-E8	Fcg RIIB	Galectin-1	B7-1
Interaction	<0.0001	0.2337	0.0456	0.3255	0.0101	0.0114
Time	<0.0001	0.0309	0.0429	0.0190	0.0014	<0.0001
Treatment	<0.0001	<0.0001	<0.0001	<0.0001	<0.0001	<0.0001

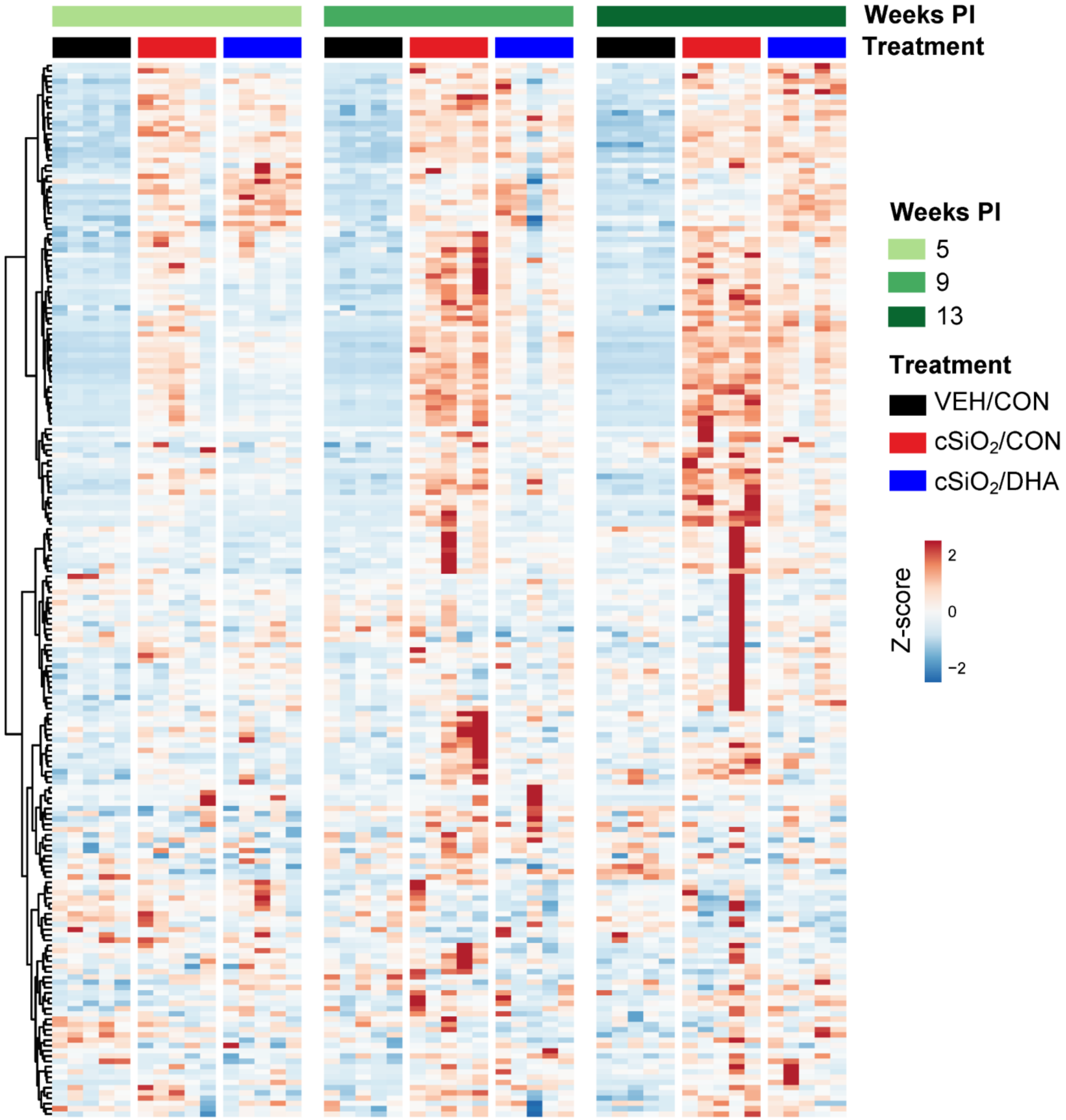
Weeks PI
Treatment
Weeks PI
5
9
13
Treatment
VEH/CON
cSiO2/CON
cSiO2/DHA
Z-score
2
0
−2

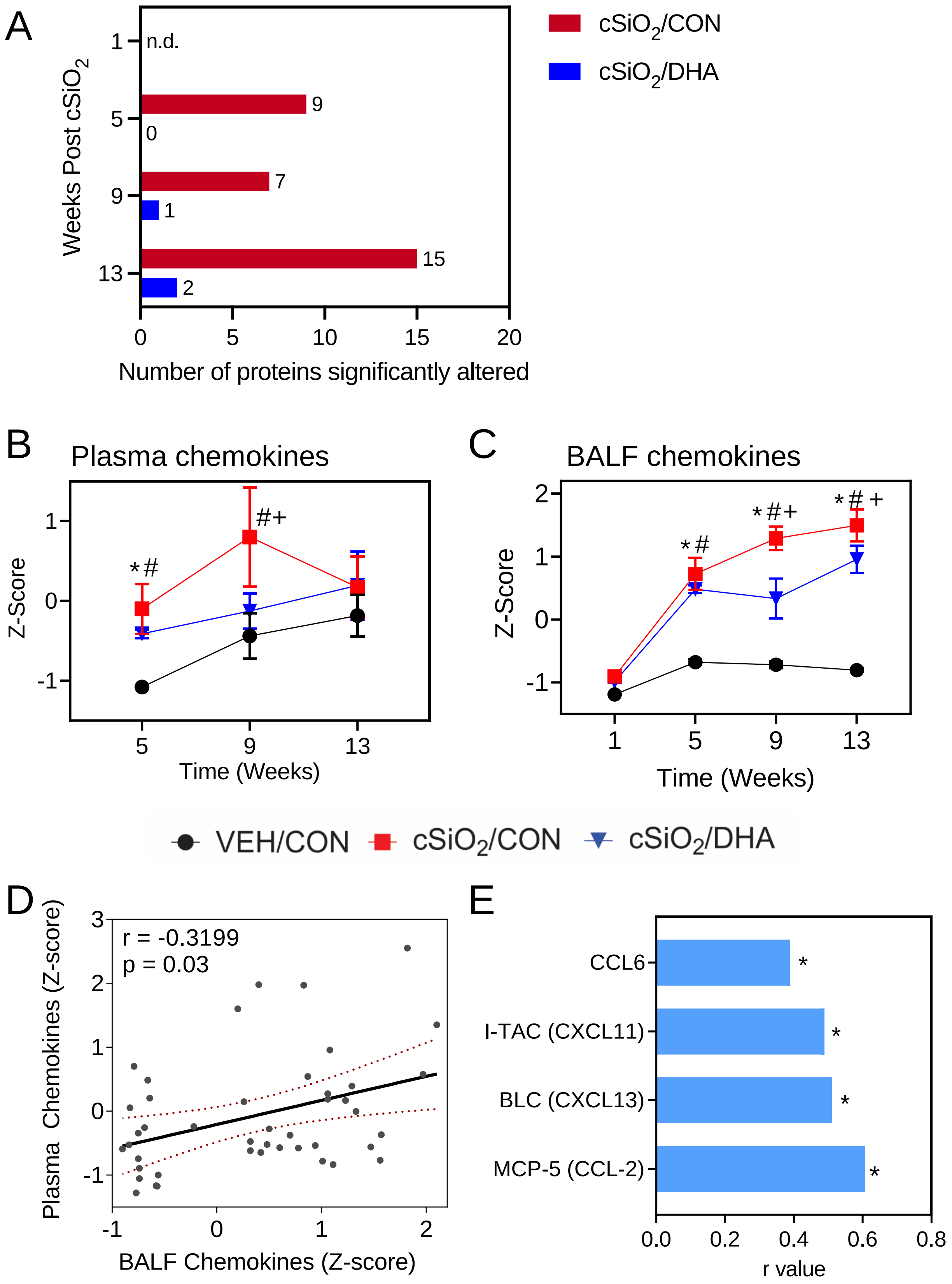
A
cSiO2/CON
cSiO2/DHA
Weeks Post cSiO2
n.d.
Number of proteins significantly altered
B
Plasma chemokines
C
BALF chemokines
Z-Score
Time (Weeks)
VEH/CON
cSiO2/CON
cSiO2/DHA
D
r = -0.3199
p = 0.03
Plasma Chemokines (Z-score)
BALF Chemokines (Z-score)
E
CCL6
I-TAC (CXCL11)
BLC (CXCL13)
MCP-5 (CCL-2)
r value
